# Male alternative reproductive tactics and sperm competition: a meta‐analysis

**DOI:** 10.1111/brv.12846

**Published:** 2022-02-28

**Authors:** Liam R. Dougherty, Michael J. A. Skirrow, Michael D. Jennions, Leigh W. Simmons

**Affiliations:** ^1^ Department of Evolution, Ecology and Behaviour University of Liverpool Crown Street Liverpool L69 7RB U.K.; ^2^ Division of Ecology & Evolution, Research School of Biology The Australian National University 46 Sullivans Creek Road Canberra ACT 2600 Australia; ^3^ Centre for Evolutionary Biology School of Biological Sciences, The University of Western Australia Crawley WA 6009 Australia

**Keywords:** alternative strategies, sperm competition, testes, spermatozoa, gonadosomatic index, ejaculate allocation, sperm quality, sneaky mating, sperm velocity, sperm motility

## Abstract

In many animal species, males may exhibit one of several discrete, alternative ways of obtaining fertilisations, known as alternative reproductive tactics (ARTs). Males exhibiting ARTs typically differ in the extent to which they invest in traits that improve their mating success, or the extent to which they face sperm competition. This has led to the widespread prediction that males exhibiting ARTs associated with a high sperm competition risk, or lower investment into traits that improve their competitiveness before mating, should invest more heavily into traits that improve their competitiveness after mating, such as large ejaculates and high‐quality sperm. However, despite many studies investigating this question since the 1990s, evidence for differences in sperm and ejaculate investment between male ARTs is mixed, and there has been no quantitative summary of this field. Following a systematic review of the literature, we performed a meta‐analysis examining how testes size, sperm number and sperm traits differ between males exhibiting ARTs that face either a high or low sperm competition risk, or high or low investment in traits that increase mating success. We obtained data from 92 studies and 67 species from across the animal kingdom. Our analyses showed that male fish exhibiting ARTs facing a high sperm competition risk had significantly larger testes (after controlling for body size) than those exhibiting tactics facing a low sperm competition risk. However, this effect appears to be due to the inappropriate use of the gonadosomatic index as a body‐size corrected measure of testes investment, which overestimates the difference in testes investment between male tactics in most cases. We found no significant difference in sperm number between males exhibiting different ARTs, regardless of whether sperm were measured from the male sperm stores or following ejaculation. We also found no significant difference in sperm traits between males exhibiting different ARTs, with the exception of sperm adenosine triphosphate (ATP) content in fish. Finally, the difference in post‐mating investment between male ARTs was not influenced by the extent to which tactics were flexible, or by the frequency of sneakers in the population. Overall, our results suggest that, despite clear theoretical predictions, there is little evidence that male ARTs differ substantially in investment into sperm and ejaculates across species. The incongruence between theoretical and empirical results could be explained if (*i*) theoretical models fail to account for differences in overall resource levels between males exhibiting different ARTs or fundamental trade‐offs between investment into different ejaculate and sperm traits, and (*ii*) studies often use sperm or ejaculate traits that do not reflect overall post‐mating investment accurately or affect fertilisation success.

## I INTRODUCTION

### (1) Background

The plainfin midshipman *Porichthys notatus* is a species of toadfish native to the Eastern Pacific Ocean. Males alone care for the offspring; females deposit their eggs into nests built by males, who defend the eggs from predators and keep them oxygenated by fanning them with their fins (Brantley & Bass, 1994). Parental males spend a significant amount of time defending their nests from rival males, and court females by producing low‐frequency hums (Brantley & Bass, 1994). However, not all males in the population pursue this parental tactic. A small proportion of males exhibit a ‘sneaking’ tactic (Brantley & Bass, 1994; Fitzpatrick *et al*., 2016). Sneaker males patrol the nests of parental males, waiting for new females to spawn there. At the exact moment of spawning, when both the female and parental male release their gametes into the nest, sneaker males attempt to ‘steal’ fertilisations by simultaneously releasing their sperm into the nest (Fitzpatrick *et al*., 2016).

Plainfin midshipman males provide a striking example of alternative reproductive tactics (ARTs). ARTs are discrete tactics or strategies performed by individuals within a sex, usually males, to obtain fertilisations (Gross, 1996; Brockmann, 2001; Oliveira, Taborsky & Brockmann, 2008), which may also involve discontinuous variation in physiological and morphological traits among individuals. For example, male ARTs often involve a dominant morph that invests heavily into attracting females and competing to repel rivals, and a sneaker morph that is much smaller and attempts to avoid such competition (Gross, 1996). ARTs are predicted to arise for one of two reasons. First, males can often benefit from avoiding the costs associated with sexual competition, or by parasitising the reproductive efforts of other males (Taborsky, 1994). In such cases, ARTs persist because males exhibiting different tactics have roughly equal fitness payoffs at equilibrium, with each tactic maintained in the population through negative frequency‐dependent selection (e.g. Gross, 1991; Shuster & Wade, 1991). There are, however, very few robust examples of tactics with equal fitness being maintained by negative frequency‐dependent selection (Gross, 1996; Oliveira *et al*., 2008). By contrast, there is strong evidence for a second explanation that males may often be unable to breed in the conventional way, for example because they are small or in poor condition and so are unlikely to outcompete rivals in a straight competition (Gross, 1996). Here, males may employ ARTs because they are ‘making the best of a bad job’ (Dawkins, 1980). In such cases, ARTs can persist in a population even if the fitness payoffs of the different tactics are not equal. Indeed, a common source of variation in competitive ability is age, especially in fishes which grow continuously throughout their life (Taborsky, 2008). Here, males may sneak when they are young and small, and switch strategies after they reach a threshold body size and become competitive (Oliveira *et al*., 2008).

Males exhibiting ARTs often face different levels of sperm competition. Sperm competition is competition between the sperm from different males for access to a female's eggs (Parker, 1970; Simmons, 2001). For species that exhibit external fertilisation (sperm and eggs meet outside of the body), sperm competition occurs when multiple males spawn with a female at the same time. For internally fertilising species (sperm and eggs meet inside the female reproductive tract), sperm competition occurs when females mate with more than one male before producing offspring. The externally fertilising plainfin midshipman males face fundamentally differing risks of sperm competition (the proportion of fertilisation opportunities in which they compete directly with a rival's sperm) depending on which ART they adopt: whereas parental males only spawn with rivals in a minority of cases, sneaker males *always* spawn in the presence of at least one parental male (Fitzpatrick *et al*., 2016). This asymmetry in the risk of sperm competition occurs in many species with sneak‐mating males (Parker, 1990b; Taborsky, 1998; Kustra & Alonzo, 2020) as well as species with other types of ARTs (see Section (2)). For species without ARTs, game‐theoretical models predict that males should increase their investment into sperm production and ejaculate size as the risk of sperm competition increases (Parker & Pizzari, 2010), and this is well supported empirically (Gage & Baker, 1991; Kelly & Jennions, 2011; Lüpold *et al*., 2020). These observations have led to the prediction, supported by formal models, that males exhibiting tactics that elevate the risk of sperm competition should invest more into sperm production, and produce larger ejaculates (Parker, 1990a,b; Gage, Stockley & Parker, 1995; Ball & Parker, 2003; Parker & Pizzari, 2010). Male ARTs may also influence investment into sperm production and ejaculates in two other important ways. First, an ART might cause a male to occupy a non‐favoured role, which will reduce his fertilisation success for reasons other than the competitiveness of his ejaculate (Parker, 1990a). For example, males in non‐favoured roles may be forced to spawn at a greater distance from females, or find that females discriminate against using their sperm (Parker, 1990a; Ball & Parker, 2003). These males can benefit by increasing the competitiveness of their ejaculate to compensate for this disadvantage. Second, males exhibiting ARTs often show reduced investment into secondary sexual traits that are used in fighting for access to females, and/or during courtship to attract females and persuade them to mate (Gross, 1996; Brockmann, 2001; Oliveira *et al*., 2008). By forgoing such investment into pre‐mating traits, males may free up resources that can be invested into post‐mating traits instead (Parker, Lessells & Simmons, 2013; Lüpold *et al*., 2014; Simmons, Lüpold & Fitzpatrick, 2017).

Males can increase their post‐mating competitiveness by producing more sperm at each mating, in order to outnumber the sperm of their rivals (Parker, 1970; Simmons, 2001; Simmons & Fitzpatrick, 2012). Increasing ejaculate size is especially beneficial when fertilisation follows the principle of a ‘fair raffle’. In such species, any given sperm has an equal chance of fertilisation, so that the more sperm that a male ejaculates, the greater the chance that one will reach an egg first (Parker & Pizzari, 2010). This principle applies to the majority of externally fertilising aquatic species, because here sperm and eggs meet randomly in the water column. In internally fertilising species, sperm may not have an equal chance of fertilisation, because the positioning of the ejaculate within the female reproductive tract can influence sperm uptake and utilisation (Simmons, 2001; Section (2)). However, in such cases males may still benefit from producing large ejaculates if this enables them to displace sperm from previous males (Parker & Simmons, 1991). A common metric used to infer investment in sperm number is testes size: larger testes have more seminiferous tissue, and so produce sperm at a greater rate (e.g. Ramm & Stockley, 2010). Indeed, there is good evidence that males in species that face a greater level of sperm competition have relatively larger testes (after controlling for body size), and that males that produce larger ejaculates tend to have greater fertilisation success (Simmons, 2001; Kelly & Jennions, 2011; Simmons & Fitzpatrick, 2012; Lüpold *et al*., 2020).

It is important to note that theoretical models of sperm competition typically distinguish between sperm/ejaculate ‘expenditure’ *versus* ‘allocation’ (Parker & Pizzari, 2010). In this context, sperm/ejaculate expenditure typically refers to long‐term investment into sperm production or sperm‐producing organs (Parker, 2016). By contrast, sperm/ejaculate allocation typically refers to investment into a single ejaculate. In other words, males produce sperm (expenditure), which are then allocated to individual matings. This distinction is important, because models suggest that optimal evolutionary strategies may differ for sperm expenditure and allocation (Parker & Pizzari, 2010), and we expand on this point in Section (2). However, these terms may have different meanings in other fields; for example, the term ‘allocation’ is often used in life‐history theory (Van Noordwijk & de Jong, 1986). Therefore, in this review we refer to specific traits whenever possible (e.g. investment into sperm production, ejaculate size, or sperm traits) in order to avoid confusion.

Males can also increase their post‐mating competitiveness by producing sperm with high fertilisation ability [i.e. high‐‘quality’ sperm (Snook, 2005; Simmons & Fitzpatrick, 2012)]. Comparative studies typically find that species with higher levels of sperm competition produce sperm that are longer and swim faster, and have ejaculates with a higher proportion of viable sperm (Snook, 2005; Simmons & Fitzpatrick, 2012; Lüpold *et al*., 2020). Within species, sperm fertilisation ability has been shown to be influenced by sperm length (Lüpold *et al*., 2012; Bennison *et al*., 2015), swimming speed (Birkhead *et al*., 1999; Gage *et al*., 2004), and viability (García‐González & Simmons, 2005), but the direction of these effects is inconsistent. For example, in some species longer sperm are better at fertilisation, whereas in other species shorter sperm are better (Simmons & Fitzpatrick, 2012). Other traits that have been suggested to affect fertilisation ability include sperm longevity (Snook, 2005), and adenosine triphosphate (ATP) content [ATP is produced by the mitochondria of sperm and provides the energy for sperm motility (Werner & Simmons, 2008; Tourmente, Varea‐Sánchez & Roldan, 2019)], with high‐quality sperm assumed to be motile for longer and with a higher ATP content. One important point to note is that sperm traits are often significantly correlated with each other, and are unlikely to evolve independently (Snook, 2005; Simmons & Fitzpatrick, 2012). These correlations may partly explain the mixed results seen in intraspecific studies (see Section IV for more discussion).

Species with male ARTs may provide the best opportunity to examine intraspecific variation in sperm and ejaculate investment, given the clear differences in post‐mating competition experienced by males using each tactic. Since this question was first investigated in the 1990s (e.g. Jennings & Philipp, 1992; Stockley *et al*., 1994; Gage *et al*., 1995), a large number of studies have compared differences in investment into sperm production and ejaculates between ARTs. A recent narrative review summarising the findings of these studies concluded that sneaker males have relatively larger testes (after controlling for body size) and produce ejaculates with a higher density of sperm when compared to non‐sneaker males, but there was no clear relationship between ARTs and any morphological sperm traits (Kustra & Alonzo, 2020). Importantly, these conclusions were based on counting the number of significant and non‐significant results reported from each study. An alternative approach is formally to quantify the direction and magnitude of statistical effects using meta‐analysis (Arnqvist & Wooster, 1995; Koricheva, Gurevitch & Mengeresen, 2013). This approach has several benefits, including: (*i*) a focus on effect sizes rather than *P* values; (*ii*) weighting of studies based on their sample size; (*iii*) formal methods to account for potential publication bias in the literature; (*iv*) the ability to test statistically for the effect of continuous or categorical moderating factors; and (*v*) the ability to control for phylogenetic non‐independence (Koricheva *et al*., 2013).

### (2) Factors influencing the relationship between ARTs and sperm investment

The recent review by Kustra & Alonzo (2020) found that the relationship between ARTs and investment into sperm production and ejaculates is variable across species, especially for sperm traits. Part of this variation may be due to the action of moderating factors that have not been investigated quantitatively. One of the strengths of meta‐analysis is the ability formally to test how potential moderators influence the differences between ARTs. In this section, we review several factors that might affect the relationship between ARTs and sperm investment.

One important consideration is the extent to which ARTs are flexible (Kustra & Alonzo, 2020). The framework of Taborsky (1998) considers three main types of ART. First, fixed tactics arise following distinct developmental trajectories, and are non‐reversible at adulthood. In this case, male expression of a tactic is based either on inherited genetic differences (e.g. Lank *et al*., 1995; Sandkam *et al*., 2021), or conditions experienced during early development. The major and minor morphs in dung beetles (Emlen, Hunt & Simmons, 2005) and the alternative male morphs in salmonids (Gross, 1985) are examples of ARTs that are fixed early in life. However, such fixed tactics are probably the exception rather than the rule (Gross, 1996; Oliveira *et al*., 2008). Second, and probably more commonly, state‐dependent (also known as sequential) tactics are conditional tactics which typically change with an individual's age, body size or condition (Gross, 1996). Males may exhibit more than one state‐dependent tactic over their lifetime, but typically only switch once, and usually in one direction (for example, from sneaking when young/small to guarding when old/large). State‐dependent tactics are common in fish, often because they grow continuously throughout their life (Taborsky, 1998). Both fixed and state‐dependent tactics are often associated with distinct male morphs. Finally, plastic (or simultaneous) tactics are fully flexible, and their use is typically unrelated to morphological differences. Males can switch tactics rapidly, and usage is often based on the immediate social or environmental context. For example, poecilid males often show a mix of consensual matings where they court females, and non‐consensual matings where they attempt to force copulations (e.g. Hurtado‐Gonzales & Uy, 2009; Smith & Ryan, 2010). Fixed tactics show the least flexibility and the highest potential for differential expenditure, and so are expected to show the greatest difference in sperm production, ejaculate size or sperm traits between ARTs. State‐dependent tactics have moderate amounts of flexibility, but the potential for specialisation in sperm production (e.g. testes size) may be limited by canalisation of gonadal traits early in life. However, state‐dependent tactics still allow for the possibility of differences in the allocation of sperm into each ejaculate. Finally, the high flexibility of plastic tactics means the potential for shifts in investment into some traits is unlikely, but still possible for others. Clearly, investment into sperm production (either through changes in testes size or morphology) cannot be significantly altered minute‐to‐minute. However, sperm traits such as motility or longevity may show more potential for flexibility over minutes or hours, especially if these effects are mediated by seminal fluid composition (e.g. Locatello, Poli & Rasotto, 2013; Poli, Locatello & Rasotto, 2018), and ejaculate size can also be modulated depending on the context (Kelly & Jennions, 2011).

Fertilisation mode could also influence investment into sperm production, ejaculate size, or sperm traits, for several reasons (Fitzpatrick, 2020). First, sperm limitation may be more of a problem for aquatic external fertilisers, because ejaculates can rapidly be diluted (Liao *et al*., 2018). Therefore, external fertilisers may be more likely to increase investment into sperm production, and produce larger ejaculates. Second, strong sperm precedence or cryptic female choice in internal fertilisers can weaken the relationship between sperm number and fertilisation success (Simmons, 2001), thus reducing the benefits of sneaking. Third, the sperm of internal and external fertilisers encounter different environments, which may favour different sperm traits. For example, faster, short‐lived sperm may be more important for some external fertilisers where sperm only need to survive for a short period, and dilution effects and water flow are more important determinants of male fertilisation success (Liao *et al*., 2018). By contrast, slower, longer‐lived sperm may be more important in internal fertilisers where sperm storage is more prevalent (Snook, 2005).

Theoretical models also highlight two important cases where ARTs should not lead to differential post‐mating investment, even when tactics differ in sperm competition risk. First, evolutionarily stable strategy (ESS) models predict that males facing a greater risk of sperm competition should increase their investment into sperm production (sperm expenditure), but not ejaculate allocation (Parker & Ball, 2005; Parker & Pizzari, 2010). ESS models predict that ejaculate allocation (i.e. ejaculate size) should be dynamically adjusted according to the immediate social environment (Parker & Pizzari, 2010). As such, the number of rivals present during a spawning is expected to be a stronger determinant of ejaculate allocation than a male's ART (Parker *et al*., 1996). This difference is not typically discussed in reviews of ARTs and sperm competition, probably because few studies in this area consider the size of, or number of sperm present in, single ejaculates (Section (2)). Another insight from game‐theoretical models is that the difference in post‐mating investment between guarders and sneakers should depend on the relative frequency of sneakers in the population (Parker, 1990b; Gage *et al*., 1995). When sneakers are rare, guarders should expend very little on sperm because they rarely face sperm competition, and sneakers should invest minimally because of the low expenditure by guarders. However, when sneakers are as common as guarders, or sneaking is involved in almost all guard matings, guarders will face as high a sperm competition risk as sneakers, and males exhibiting both tactics are expected to invest equally into sperm and ejaculates. These models lead to the prediction that the disparity in post‐mating investment between guarders and sneakers should be highest when the risk of sneaking is at an intermediate level (Parker, 1990b; Gage *et al*., 1995). However, empirical support for this prediction is lacking: while a comparison of 16 dung beetle species showed that species with a larger proportion of minor males had relatively larger testes (after correcting for body size), the disparity in relative testes size between major and minor males did not relate to minor male frequency (Simmons, Emlen & Tomkins, 2007).

Finally, methodological issues can complicate measurement of the relationship between ARTs and investment into sperm production, ejaculate size or sperm traits. For example, testes size is often compared between ARTs using the proportion of body tissue accounted for by the testes, especially in fishes. This measure is known as the gonadosomatic index (GSI), and is calculated as 100× (testes mass/soma mass) (Devlaming, Grossman & Chapman, 1982). This metric has been criticised as inappropriate for comparing males exhibiting different ARTs, because it only ‘controls’ for male body size when testes size scales isometrically with body size (the slope of the relationship between testes size and body size is exactly 1; Tomkins & Simmons, 2002). When the relationship between body size and testes size is not isometric (either because the slope differs from 1, the intercept differs from 0, or both), spurious results will be obtained. For example, a slope of less than 1 (negative allometry) will result in smaller individuals having a higher GSI, independent of any investment differences between male tactics (Simmons, Tomkins & Hunt, 1999; Tomkins & Simmons, 2002). This approach is further problematic because it assumes that testes allometry is the same for each male tactic, which is unlikely in species with clear morphological differences between tactics (Tomkins & Simmons, 2002). For both of these reasons, the use of GSI is likely to overestimate differences in investment into sperm production between male tactics.

### (3) Meta‐analysis overview

We systematically searched the literature for studies comparing sperm investment or sperm traits between males of the same species exhibiting two or more ARTs that are expected to differ in (*i*) sperm competition risk, or (*ii*) the degree of investment into traits that increase mating success. Our searches resulted in three separate data sets, consisting of effect sizes examining the relationship between male ARTs and: (*1*) testes size; (*2*) sperm quantity; and (*3*) sperm traits. Notably, the sperm quantity data set included estimates representing both sperm expenditure (the number of sperm present in dissected testes) and sperm allocation (the number of sperm present in ejaculates). For each data set we performed a phylogenetically controlled meta‐analysis comparing males exhibiting tactics that face a high or a low sperm competition risk, or have a high or low investment into secondary sexual traits that are used in fighting for access to females, and/or during courtship to attract females and persuade them to mate. We also use this framework to test quantitatively for factors moderating the strength and direction of the relationship between sperm investment and ARTs. We have six main predictions:

1. Males exhibiting ARTs that elevate sperm competition risk, or who invest less into traits that increase mating success, will invest more into sperm production, produce larger ejaculates per mating, and produce more competitive sperm (sperm that are longer, swim faster, stay motile for longer or have a higher ATP content) or ejaculates (containing a high proportion of viable or motile sperm).
2. ARTs will differ in the average number of sperm present in the testes (sperm expenditure) but not in the average number of sperm ejaculated (sperm allocation), because the latter is likely to be more strongly influenced by the immediate social environment.
3. The difference in investment into sperm production (sperm expenditure) between ARTs will be greater for species in which male tactics are fixed for life than those in which male tactics are sequentially or fully flexible.
4. The difference in sperm investment into sperm production, ejaculate size and sperm traits between ARTs will be greater for external fertilisers than internal fertilisers because fertilisation is likely less constrained by interactions between sperm and the female reproductive tract.
5. The difference in investment into sperm production, ejaculate size and sperm traits between ARTs will be negatively related to the proportion of sneakers in the population.
6. The difference in testes size between ARTs will be greatest for studies measuring the GSI than those using other metrics.

## II METHODS

Throughout we follow the recent extension to the PRISMA reporting guidelines for ecology and evolutionary biology by (O'Dea *et al*., 2021). See the online Supporting Information, Appendix S1, for a completed PRISMA checklist.

### (1) Systematic searches

We focused our searches on published, peer‐reviewed studies. We searched for published papers in three ways. First, we searched the online database *Web of Science* for papers using a variety of key words relating to ARTs and sperm investment. We searched all years and all databases available in the *Web of Science* Core Collection. Nineteen separate searches were performed, using the following terms:

1. ‘alternative mating’ AND (sperm* OR ejaculat*);
2. ‘alternative mating’ AND (testes OR testis OR gonad*);
3. ‘alternative reproductive’ AND (sperm* OR ejaculat*);
4. ‘alternative reproductive’ AND (testes OR testis OR gonad*);
5. (sneak* OR satellite* OR helper OR guard*) AND (sperm* OR ejaculat*);
6. (sneak* OR satellite* OR helper OR guard*) AND (testes OR testis OR gonad*);
7. (parr* OR jack*) AND (sperm* OR ejaculat*);
8. (parr* OR jack*) AND (testes OR testis OR gonad*);
9. sneak* AND (sperm* OR ejaculat*);
10. guard* AND (sperm* OR ejaculat*);
11. satellite* AND (sperm* OR ejaculat*);
12. helper AND (sperm* OR ejaculat*);
13. parr* AND (sperm* OR ejaculat*);
14. jack* AND (sperm* OR ejaculat*);
15. sneak* AND (testes OR testis OR gonad*);
16. guard* AND (testes OR testis OR gonad*);
17. helper AND (testes OR testis OR gonad*);
18. parr* AND (testes OR testis OR gonad*);
19. jack* AND (testes OR testis OR gonad*).

Second, we conducted reverse searches of papers citing nine influential articles in this area, again using *Web of Science*. We searched for papers citing Gage & Baker (1991), Gage *et al*. (1995), Neff, Fu & Gross (2003), Parker (1990b), Parker *et al*. (2013), Simmons *et al*. (2007), Simmons *et al*. (1999), Taborsky (1994) and Taborsky (1998). Third, we read all the papers identified in the recent narrative review of male ARTs and sperm competition (Kustra & Alonzo, 2020). We also obtained one data set prior to publication (Loveland, Lank & Küpper, 2021) after contacting the authors regarding another paper.

Searches were performed in two stages. Initially we conducted key word searches on 7 December 2018 and reverse searches on 15 January 2019. In the second stage, both key word and reverse searches were conducted on 22 October 2020, in order to cover 2019 and 2020 (only six new papers were found in the second stage). All searches during the first stage were performed by M.J.A.S., and in the second stage by L.R.D. The results of the searches, plus the screening process, are outlined in Fig. S1. In total, our literature searches identified 3861 studies. Search results were imported into the web application Rayyan (Ouzzani *et al*., 2016), and the titles and abstracts screened for eligibility. Title and abstract screening identified 263 potentially eligible studies, which were then downloaded and read in their entirety.

### (2) Study inclusion criteria

To be considered eligible for inclusion, a study had to compare sperm traits between males of the same species exhibiting discrete ARTs. We did not consider female ARTs. To be considered an ART, males had to show discrete reproductive tactics or morphs (e.g. a bimodal distribution in body size), or exhibit behaviours that could be assigned to mutually exclusive categories (e.g. consensual *versus* coercive mating). We excluded studies relating sperm traits to continuous variation in any male phenotype (e.g. body size, ornament/weapon size). We also excluded studies of species where subordinates are reproductively suppressed by dominants (e.g. Fitzpatrick *et al*., 2006; Kustan, Maruska & Fernald, 2012), and studies of sequential hermaphrodites.

We included three types of ARTs, based on the categorisation by Taborsky (1998):

1. Fixed tactics. Tactics were assigned to this category if they have distinct developmental trajectories, and are non‐reversible at adulthood.
2. Sequential (state‐dependent) tactics. Tactics were assigned to this category if their expression is conditional on any aspect of individual state, such as age, body size or condition. Tactics were also assigned to this category if they are associated with clear morphological differences, but cannot be linked either to genetic differences or distinct developmental trajectories between male morphs.
3. Flexible tactics. Tactics were assigned to this category if they are fully reversible, and not associated with alternative morphologies.

We focused only on ARTs that could potentially influence male sperm competition risk, or that differed clearly in investment into traits that increase mating success. The actual risk of sperm competition is rarely quantified for either male tactic, so we primarily relied on behavioural observations or assertions made by the study authors. We excluded species with ARTs that are unlikely to differ in sperm competition risk, such as the burrowing bee *Amegilla dawsoni* for which observational and genetic data suggest that females only ever mate once (Simmons, Tomkins & Alcock, 2000). We collected data for 18 types of ARTs (Table 1).

**Table 1:** Overview of the 18 alternative reproductive tactics (ARTs) included in the data set, with a description of how each is predicted to influence investment into sperm or ejaculate traits

Species	ARTs	Reason for inclusion	Positive effect size
**Roach** *Rutilus rutilus*	Attractive *vs* unattractive	Unattractive males face greater SC risk	Unattractive > attractive
**Golden julie** *Julidochromis ornatus*	Breeder *vs* cooperative breeder	Cooperative breeders face greater SC risk	Cooperative breeder > breeder
**Masked julie** *Julidochromis transcriptus*			
**Golden julie** *Julidochromis ornatus*	Breeder *vs* helper	Helpers face greater SC risk	Helper > breeder
**Masked julie** *Julidochromis transcriptus*			
**Red‐backed fairy wren** *Malurus melanocephalus*			
**Chinook salmon** *Oncorhynchus tshawytscha*	Dominant *vs* subordinate	Subordinates face greater SC risk	Subordinate > dominant
**Dunnock** *Prunella modularis*			
**Arctic char** *Salvelinus alpinus*			
**Ant** *Cardiocondyla obscurior*	Fighter *vs* disperser	Dispersers face greater SC risk	Disperser > fighter
**Atlantic horseshoe crab** *Limulus polyphemus*	Guarder *vs* satellite	Satellites face greater SC risk	Satellite > guarder
**Quacking frog** *Crinia georgiana*	Guarder *vs* sneaker	Sneakers face greater SC risk	Sneaker > guarder
**Slender inshore squid** *Doryteuthis plei*			
**European earwig** *Forficula auricularia*			
**Black goby** *Gobius niger*			
**Wellington tree weta** *Hemideina crassidens*			
**Spear squid** *Heterololigo bleekeri*			
**Longear sunfish** *Lepomis megalotis*			
**Masu salmon** *Oncorhynchus masou*			
**Chinook salmon** *Oncorhynchus tshawytscha*			
**Plainfin midshipman** *Porichthys notatus*			
**European bitterling** *Rhodeus amarus*			
**Peacock blenny** *Salaria pavo*			
**Atlantic salmon** *Salmo salar*			
**Brown trout** *Salmo trutta*			
**Harvestman** *Serracutisoma proximum*			
**Bluehead wrasse** *Thalassoma bifasciatum*			
**Grass goby** *Zosterisessor ophiocephalus*			
**Dung beetle** *Lethrus apterus*	Major *vs* minor	Minors face greater SC risk	Minor > major
**Dung beetle** *Onthophagus aeruginosis*			
**Dung beetle** *Onthophagus alcyonides*			
**Dung beetle** *Onthophagus australis*			
**Dung beetle** *Onthophagus binodis*			
**Dung beetle** *Onthophagus cribripennis*			
**Dung beetle** *Onthophagus fuliginosus*			
**Dung beetle** *Onthophagus gazella*			
**Dung beetle** *Onthophagus haagi*			
**Dung beetle** *Onthophagus hecate*			
**Dung beetle** *Onthophagus nigriventris*			
**Dung beetle** *Onthophagus nodulifer*			
**Dung beetle** *Onthophagus rupicapra*			
**Dung beetle** *Onthophagus sloanei*			
**Dung beetle** *Onthophagus taurus*			
**Dung beetle** *Onthophagus vermiculatus*			
**Seba's short‐tailed bat** *Carollia perspicillata*	Harem *vs* sneaker	Sneakers face greater SC risk	Sneaker > harem
**Cichlid** *Neolamprologus mondabu*			
**Dunnock** *Prunella modularis*	Monogamous *vs* polyandrous	Polyandrous males face greater SC risk	Polyandrous > monogamous
**Dusky frillgoby** *Bathygobius fuscus*	Nesting *vs* sneaker	Sneakers face greater SC risk	Sneaker > nesting
**Cichlid** *Lamprologus callipterus*			
**Cichlid** *Lamprologus lemairii*			
**Sand goby** *Pomatoschistus minutus*			
**Molly Miller** *Scartella cristata*			
**Corkwing wrasse** *Symphodus melops*			
**Ocellated wrasse** *Symphodus ocellatus*			
**Tree swallow** *Tachycineta bicolor*	Paired *vs* extra‐pair	Extra‐pair males face greater SC risk	Extra‐pair > paired
**Cichlid** *Amatitlania siquia*	Parental *vs* sneaker	Sneakers face greater SC risk	Sneaker > parental
**Three‐spined stickleback** *Gasterosteus aculeatus*			
**Pumpkinseed** *Lepomis gibbosus*			
**Bluegill** *Lepomis macrochirus*			
**Round goby** *Neogobius melanostomus*			
**Cichlid** *Telmatochromis temporalis*			
**Cichlid** *Telmatochromis vittatus*			
**Common shrew** *Sorex araneus*	Resident *vs* searcher	Searchers face greater SC risk	Searcher > resident
**Bluenose shiner** *Pteronotropis welaka*	Territorial *vs* non‐territorial	Non‐territorial males face greater SC risk	Non‐territorial > territorial
**Cortez triplefin** *Axoclinus nigricaudus*	Territorial *vs* sneaker	Sneakers face greater SC risk	Sneaker > territorial
**Painted dragon** *Ctenophorus pictus*			
**Carmine triplefin** *Enneanectes carminalis*			
**Ruff** *Calidris pugnax*	Territorial *vs* female mimic	Female‐mimics invest less in courtship	Female mimic > territorial
**Melanzona guppy** *Poecilia parae*	Consensual *vs* coercive matings	Coercive males invest less in courtship	Coercive > consensual
**Guppy** *Poecilia reticulata*			
**Panuco swordtail** *Xiphophorus nigrensis*			

SC, sperm competition.

We considered three categories of post‐mating traits.

1. *Testes size*. We included studies estimating both the mass and volume of the sperm‐producing organs, as a proxy for investment into sperm production. Ideally, we only included data on relative testes size, after controlling for body size. However, we also used absolute testes size as a metric when there was no significant difference in body size between male tactics (Stockley *et al*., 1994; Peer, Robertson & Kempenaers, 2000; Olsson *et al*., 2009). Studies controlled for body size using (*i*) the GSI, (*ii*) the residuals of a regression of body size against testes size (e.g. Simmons *et al*., 2007), or (*iii*) analysis of covariance (ANCOVA) (Tomkins & Simmons, 2002). Most studies used body mass as a measure of body size, although we also included studies in insects using pronotum or leg length as a proxy for body size (Kelly, 2008; Rosa *et al*., 2019).
2. *Sperm quantity*. We included data on the number of sperm cells present in the ejaculate or packaged into a spermatophore (sperm allocation), or present in the testes after stripping of live males or dissection of dead males (sperm expenditure). Ejaculates were stripped from live males either by applying gentle pressure to the abdomen or testes, or by electrostimulation (e.g. Sasson, Johnson & Brockmann, 2015; Meniri *et al*., 2019). After collection of the ejaculate, sperm quantity was estimated by counting the number or density of sperm cells in a given volume of ejaculate, calculating the volume of the ejaculate (e.g. Simmons *et al*., 1999), or measuring the length of the spermatophore (Apostólico & Marian, 2017).
3. *Sperm traits*. We collected sperm traits (morphology, physiology, or behaviour) which are purported to relate to sperm competitiveness. In all but two cases (Simmons *et al*., 1999; Apostólico & Marian, 2018) sperm traits were measured using sperm that had not been ejaculated or packaged into a spermatophore.
  - Average sperm length. All identified studies focused on flagellate sperm, which swim using a ‘tail’, or ‘flagellum’. The flagellum is usually the longest component of the sperm cell, so in all cases we used data on either total cell length or flagellum length. When multiple components were reported, we used flagellum length only.
  - Average sperm swimming speed. Speed is estimated using either manual or automated computer‐assisted sperm analysis video analysis. There are multiple ways to estimate swimming speed provided by common video analysis packages (Sloter *et al*., 2006), with the most common being curvilinear velocity (*V*_CL_, the velocity across the track taken by the cell between each frame). Other measures include linear velocity (*V*_SL_: velocity in a straight line between the first and last frame), and average path velocity (*V*_AP_; a smoothed version of *V*_CL_). These measures are usually highly correlated within studies. One study also used flagellum beat frequency to calculate swimming speed (Butts *et al*., 2017). When multiple speed estimates were available, we used *V*_CL_.
  - Sperm longevity. Studies measured sperm longevity as either: (*i*) the time until all (or a high proportion of) sperm stopped moving forward; (*ii*), the time when the average swimming speed of sperm fell below some defined value (Taborsky *et al*., 2018); (*iii*) the proportion of sperm still swimming after a defined duration (e.g. Hettyey & Roberts, 2005, 2007); or (*iv*) the slope in the decline in sperm motility over time (e.g. Fasel *et al*., 2017).
  - Sperm ATP content. ATP content is estimated by measuring the amount of light produced by the bioluminescent luciferin–luciferase reaction, which only occurs in the presence of ATP (Lundin, 2000).
  - The proportion of sperm in the ejaculate that are motile or alive. Motile sperm are those that show some degree of forward movement, and viable sperm are determined using a range of methods which differentially stain alive *versus* dead sperm (Holman, 2009). Given that relatively few studies measured sperm viability (Locatello *et al*., 2007; Smith & Ryan, 2010; Rowe *et al*., 2010; Smith, 2012; Schrempf *et al*., 2016; Green *et al*., 2020), we combined these two measures into a single category.

We excluded studies presenting other reproductive traits that do not relate directly to sperm investment, such as spermatophore morphology (e.g. Iwata, Sakurai & Shaw, 2015) or male internal reproductive anatomy (e.g. accessory gland size: Barni, Mazzoldi & Rasotto, 2001). We also excluded estimates of the fertilisation success of different male ARTs (e.g. Carroll, 1993; Adreani, 2012).

Finally, to be included in the data set a study had to present sufficient data (including sample sizes for each male tactic) for an effect size and its variance to be calculated (Section (3)).

### (3) Effect size calculations

We used the standardised mean difference, also known as Hedges' *d*, as our measure of effect size (Hedges & Olkin, 1985). This is very commonly used as an effect size when the aim is to compare average values between two groups (Nakagawa & Santos, 2012), and is especially appropriate when the two groups come from observational data (i.e. there are no control and treatment groups). We assigned effect sizes a positive direction when investment into sperm traits was higher for males exhibiting tactics associated with a greater sperm competition risk or a reduced investment into pre‐mating sexual traits (Table 1). The latter condition was relevant for males that engage in coercive matings (Pilastro & Bisazza, 1999; Hurtado‐Gonzales & Uy, 2009; Smith & Ryan, 2010; Smith, 2012) and males that exhibit female‐mimicking plumage (Loveland *et al*., 2021), which either have reduced sexual ornaments or do not court females. Following the sperm competition literature, we assumed that higher investment into post‐mating traits should result in larger testes, more sperm in the testes, more sperm in the ejaculate, a higher proportion of motile sperm in the ejaculate, and sperm that are longer, swim faster, stay motile for longer or have a higher ATP content. We note that there may be functional or resource‐allocation trade‐offs among sperm traits. For example, studies have recorded a negative within‐species correlation between sperm swimming speed and sperm longevity (Levitan, 2000; Yamamoto *et al*., 2017; Taborsky *et al*., 2018), and between sperm length and sperm longevity (Gage *et al*., 2002). However, such trade‐offs are far from universal (Snook, 2005), and the traits that are important for male fertilisation success differ across species (Simmons & Fitzpatrick, 2012). For both of these reasons we did not attempt to model trade‐offs directly; rather we assumed that all sperm traits could potentially differ between ARTs. However, we also test for widespread trade‐offs in the analysis, by comparing the average effect size for each sperm trait separately.

We obtained effect sizes from papers in one of three ways. First, we calculated the standardised mean difference directly from reported means and variances (standard deviation or standard error), using the equations in Koricheva *et al*. (2013, p. 200). These data were either taken directly from values reported in the text or tables, or extracted manually from bar plots using the online tool WebPlotDigitizer v4 (https://apps.automeris.io/wpd/). Second, we converted the results of appropriate statistical tests into the standardised mean difference using the conversion equations in Koricheva *et al*. (2013, pp. 200–201). We used results from *t*‐tests, paired *t*‐tests, and Mann–Whitney *U* tests. Finally, we performed supplementary analyses when we had access to the raw data. Raw data were either obtained from available Supporting Information, extracted manually from scatter plots using WebPlotDigitizer, or obtained by contacting the study authors (we received data from five studies in this way). In species with more than two ARTs, we performed multiple pairwise comparisons. Full information regarding effect size calculations is provided in Table S1. In cases where sperm traits (e.g. motility) were measured at multiple time points, we only considered the first time point. We extracted all available effect sizes from a study. This often resulted in multiple effect sizes per study, especially when studies reported multiple sperm traits from the same sample of individuals, which we controlled for statistically (Section (6)). All data extraction was performed by L.R.D.

Testes size is often compared between ARTs using the proportion of body tissue accounted for by the testes, especially in fishes. This measure is known as the GSI. This metric has been criticised as not accounting fully for body size (see Section (2)). Therefore, whenever possible we re‐analysed raw data on testes mass using the ANCOVA method suggested by Tomkins & Simmons (2002). For this method, we performed an ANCOVA with testes mass as the dependent variable, male tactic as the independent variable, and soma mass (body mass–testes mass) as a covariate. If body mass was measured before testes were dissected, we calculated soma mass manually. For the ANCOVA, we first ran a full model testing the effect of soma mass, male tactic, and their interaction, on testes mass. If the interaction term was not significant, this suggests that testes allometry does not differ between the male tactics. This was the case in 39 out of 44 analyses. When the interaction term was not significant, we dropped it from the model, and calculated partial eta‐squared for the fixed effect of male tactics using the EtaSq function in the R package DescTools. Partial eta‐squared was then converted to Cohen's *d* using the equation in Cohen (1988, p. 284), and Cohen's *d* was converted into Hedges' *d* using the equation in Borenstein *et al*. (2009). We used this ANCOVA approach on approximately half of the studies reporting GSI (34 of 64 effect sizes).

Studies sometimes reported non‐significant results without providing information about the direction of the effect. These effect sizes are traditionally excluded from meta‐analysis; however, this systematically biases the data set against non‐significant results. Therefore, we assigned relevant directionless effect sizes a value of zero (15 effect sizes: one testes size trait, four sperm quantity traits, 10 sperm traits), and ran the analyses with and without including these extra data points as a form of sensitivity analysis (Harts, Booksmythe & Jennions, 2016; Booksmythe *et al*., 2017; Dougherty, 2021).

### (4) Phylogeny

We estimated the phylogenetic relationships among the species in our data set in order to control for the potential non‐independence of effect sizes due to shared evolutionary history (Hadfield & Nakagawa, 2010; Koricheva *et al*., 2013). As no single phylogenetic tree was available that included all species, we constructed a supertree from available phylogenetic and taxonomic information using the Open Tree of Life (OTL) database (Hinchliff *et al*., 2015), and the rotl (Michonneau, Brown & Winter, 2016) and ape (Paradis, Claude & Strimmer, 2004) R packages. We also manually searched for phylogenetic information for species or taxa not listed in the OTL database. For the position of Opilliones in relation to arthropods, we used Giribet, Edgecombe & Wheeler (2001). The relationships among the 15 *Onthophagus* species was found in Emlen *et al*. (2005). We were unable to find information about the phylogenetic position of two species: *Onthophagus nodulifer* and *Onthophagus rupicapra*. Based on the geographic distribution of these species, and the tree in Emlen *et al*. (2005), we added both species as a polytomy at the base of the Australian *Onthophagus* clade. These two species were only present in the testes size data set. We therefore tested the sensitivity of the overall meta‐analytic mean estimate by running this model with and without the inclusion of these two species. As the supertree lacks accurate branch lengths, lengths were first set to 1 and then made ultrametric using Grafen's method (Grafen, 1989). The tree was then converted into a variance–covariance matrix for incorporation into the meta‐analysis models. For analyses including subsets of the data, we used an appropriately pruned tree (Figs S2–S4).

### (5) Moderator variables

For each study, we collected data on a range of moderator variables predicted to influence the mean effect size (see Section (2) for discussion):

1. *Taxonomic group*. We obtained data from nine taxonomic groups: cephalopods, chelicerates, arachnids, insects, fish, amphibians, reptiles, birds, and mammals. However, over 70% of effect sizes came from fish (182 out of 251), and most of the remaining groups contained few examples. Therefore, to increase our statistical power, we sorted species into three categories: invertebrates (arachnids, cephalopods, chelicerates, and insects), fish, and other vertebrates (amphibians, birds, mammals, and reptiles). We had no directional prediction based on this categorisation.
2. *Mode of fertilisation*. We obtained data for both externally and internally fertilising species. We predicted that the difference in sperm traits would be greatest for externally fertilising species, primarily because strong sperm precedence or cryptic female choice in internal fertilisers might weaken the relationship between sperm number and fertilisation success, thus reducing the benefits of sneaking.
3. *Tactic type*. We classified ARTs into one of three categories: fixed, state‐dependent, or plastic. However, for all three data sets we obtained very few estimates for plastic tactics (1–10 effect sizes per data set). Therefore, for two of the data sets (testes size and sperm quantity) we only compared fixed and state‐dependent categories (five effect sizes removed in total). We predicted that the difference in post‐mating investment would be greatest for species with fixed ARTs, because fixed tactics are set early in life and so show the highest potential for differences in post‐mating investment.
4. *Measurement*. For the testes and sperm quantity data sets, we tested whether the mean effect size differed depending on the measurement method used. For testes size, we compared estimates obtained using the GSI and relative testes size (controlling for body size; we excluded three effect sizes derived from absolute testes size for this comparison). We predicted that studies using the GSI would result in a larger effect size than those using other measures of testes investment, because this method inadequately controls for testes allometry and could lead to a spurious difference between alternative male tactics. For sperm quantity, we compared measures of sperm number, sperm volume and sperm density (we excluded a single study measuring spermatophore size from this comparison). We had no directional prediction for this category.
5. *Sperm trait*. For the sperm traits data set, we compared measures of sperm length, sperm swimming speed, sperm longevity, sperm ATP content, and the proportion of motile sperm in the ejaculate. While some studies have suggested the presence of trade‐offs between different sperm traits (e.g. between swimming speed and longevity: Levitan, 2000), such trade‐offs are not ubiquitous (Snook, 2005), and there is evidence that all of the traits may positively influence fertilisation success (Snook, 2005; Simmons & Fitzpatrick, 2012). Therefore, we had no clear directional prediction for whether some sperm traits would differ more strongly between ARTs than others.
6. *Sneaker frequency*. We searched for published estimates of the frequency of sneaker males for species showing fixed or state‐dependent tactics (the frequency of sneakers is not relevant for species exhibiting fully flexible tactics). We excluded estimates when sampling was not random with respect to male tactic. Ideally, we used demographic data from the same experimental population as the effect size. When this was unavailable, we used estimates taken from the same population, location or species (listed in order of priority). The sources for these data are listed in Table S2. We obtained data on sneaker frequency for 54 of the 67 species in our data set (Fig. S5). Following the models by Parker (1990b) and Gage *et al*. (1995), we predicted that the difference in post‐mating investment between sneaker and non‐sneaker males would be greatest when the proportion of sneakers in the population was intermediate. This is because males exhibiting both tactics are expected to invest little into sperm traits when the risk of sperm competition is very low (when there are few sneakers), and to invest highly when the risk of sperm competition is high (when there are many sneakers). In other words, we predict the average effect size to be significantly positive at intermediate sneaker frequency, and close to zero when the proportion of sneakers in the population is very high or very low.
7. *Sperm allocation* versus *expenditure*. For the sperm quantity data set, we compared estimates obtained from sperm in the ejaculate or packaged into a spermatophore (sperm allocation), or in the testes after stripping from live males or dissection of dead males (sperm expenditure). We predicted that sperm expenditure would be significantly greater for sneaker males (a significantly positive effect size), but that ARTs would not differ in terms of sperm allocation (effect size does not differ from zero) as this is more strongly influenced by the immediate social environment during mating (see Section (3)).

### (6) Statistical analysis

Our systematic searches resulted in three data sets (all data and code used in the analysis are available at 10.6084/m9.figshare.19174604), focusing on: (*1*) testes size; (*2*) sperm quantity; and (*3*) sperm traits, which we analysed separately using R v4.0.3 (R Development Core Team, 2020) and the Metafor package v2.4 (Viechtbauer, 2010). We first determined the overall mean effect size estimate using multi‐level random effects models (Nakagawa & Santos, 2012) using the rma.mv function. Each model included phylogeny, species, study ID, and observation ID as random factors. Observation ID represents the observational or residual variance, and needs to be explicitly modelled in a meta‐analytic model (Moran *et al*., 2020). Study ID was included because some studies provided multiple effect sizes (especially for the sperm traits data set). Species was included because estimates were available from more than one study for some species. The phylogeny was incorporated into all models using a variance–covariance matrix. We considered an effect size to differ significantly from zero when the 95% confidence intervals (CIs) do not overlap zero. We ran these models with and without inclusion of directionless effect sizes (Section (3)). We calculated heterogeneity across each data set using the *I* ^2^ statistic (Higgins *et al*., 2003). We also partitioned heterogeneity with respect to each of the four random factors, using the method of Nakagawa & Santos (2012). *I* ^2^ values of 25, 50 and 75% are considered low, medium and high, respectively (Higgins *et al*., 2003).

Studies often presented measures of multiple sperm traits using the same sample of males. If these traits are correlated the effect size estimates are not independent, and a meta‐analysis that does not take this into account can underestimate the uncertainty in the overall effect size estimate (Noble *et al*., 2017). We attempted to control for this potential non‐independence statistically by using a variance–covariance matrix to specify the correlation between effect sizes from the same experiment (Noble *et al*., 2017). To do this, we first created a new factor called ‘experiment ID’, with effect sizes derived from the same sample of males given the same ID code. We then produced a variance–covariance matrix specifying the correlation between each effect size in the data set. When the correlation between traits is unavailable, studies typically assume a correlation of 0.5, which is halfway between no correlation and a perfect correlation of 1 (e.g. Moran *et al*., 2020; Dougherty, 2021). Therefore, to test the sensitivity of our analysis (e.g. Bishop & Nakagawa, 2021) we produced three matrices, with effect sizes from the same experiment assumed to have a correlation of 0.25, 0.5 or 0.75. We then ran the same multi‐level random effects model as above, with the addition of experiment ID as a random effect, and study variance specified by one of the covariance matrices. We only used this approach for the sperm traits data set, because presentation of multiple correlated traits is not a feature of the testes size or sperm quantity data sets.

We used meta‐regression models to examine the effect of our moderator variables on the mean effect size (Nakagawa & Santos, 2012). Each model included phylogeny, species, study ID, and observation ID as random factors as before, but now also included one of the seven moderator variables listed in Section (5) as a categorical (taxonomic group, mode of fertilisation, tactic type, measurement, sperm trait, and sperm allocation *versus* expenditure) or continuous (sneaker frequency) fixed effect. We first tested for a quadratic relationship between sneaker frequency and the difference between ARTs, as theory predicts the difference between tactics should be greatest at intermediate sneaker frequencies (Parker, 1990b; Gage *et al*., 1995). If there was no significant quadratic effect, we also tested for a linear effect. To test whether the mean effect size differed significantly between moderator categories, we used the *Q*_M_ statistic, with a significant value indicating that the moderator accounts for a significant proportion of the between‐study heterogeneity (Koricheva *et al*., 2013). We also ran these models with the intercept term dropped to obtain estimates of the mean effect size for each categorical moderator level (in effect running a separate meta‐analysis for each factor level). All meta‐regressions were tested including directionless effect sizes. To improve our ability to detect biologically relevant differences, we excluded any trait categories with fewer than five effect sizes when performing meta‐regressions.

For the testes size data set, we also explicitly tested whether the use of the GSI could inflate the differences between male tactics in fish, in two ways. First, we estimated the mean effect size for the subset of fish studies that did not use the GSI. Second, we searched for raw testes allometry data, in order to compare directly effect size estimates from the same males derived from ANCOVA and GSI approaches. We found raw data for testes allometry for 18 out of 51 studies. We tested whether these two approaches resulted in significantly different effect size estimates using a paired *t*‐test comparing the Hedges' *d* values (*N* = 35 comparisons and 30 species).

We searched for two signs of publication bias. First, we tested for evidence of publication bias against non‐significant results. One outcome of this type of publication bias is a significant relationship between effect size and study variance, driven by ‘missing’ effect sizes of small effect and with small sample sizes (a ‘small study effect’: Koricheva *et al*., 2013). We tested for this relationship using a meta‐regression with the inverse standard error (also known as study precision) as a fixed factor, and phylogeny, species, study ID, and observation ID as random factors. Second, we tested for a change in the average effect size over time, which could reflect a change in the speed with which certain types of studies are published (Jennions & Møller, 2002). This could arise if studies with non‐significant results are less likely to be published when a research field is young. We tested for a temporal trend in effect sizes using a meta‐regression with publication year as a fixed factor, and phylogeny, species, study ID, and observation ID as random factors.

## III RESULTS

### (1) Testes size

**Fig. 1:**
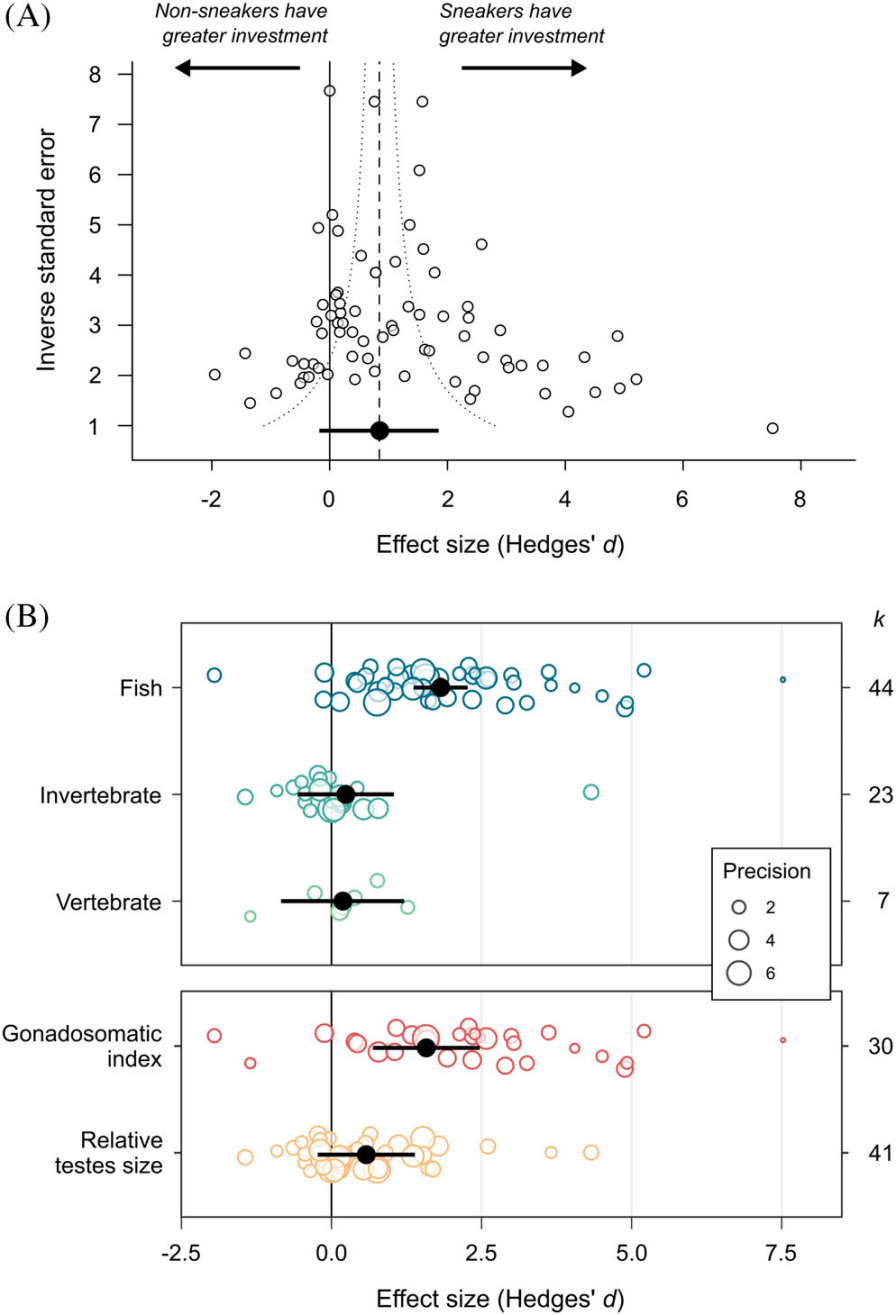
Difference in testes size (Hedges' *d*) between male alternative reproductive tactics in relation to (A) study variance, and (B) taxonomic group (top panel) and size measure (bottom panel). In (A), the dashed vertical line represents the meta‐analytic mean, and the dotted lines are the 95% pseudo‐confidence interval. In (B), points are scaled according to study variance (precision). In all panels, black points represent the meta‐analytic mean, and black bars show the 95% confidence interval. *k* = number of effect sizes for each category.

The testes size data set consisted of 74 effect sizes from 51 studies and 53 species. Over half of the effect sizes came from fish (44 effect sizes, 28 species). We obtained sneaker frequency data for 45 species in this data set. Overall, there was no significant difference in investment in testes size between male ARTs (mean *d* = 0.87, 95% CI = −0.16 to 1.90, *k* = 74; Fig. 1A). This remained the case after removing the one directionless effect size (mean *d* = 0.90, 95% CI = −0.15 to 1.95, *k* = 73), and after removing the two *Onthophagus* species with uncertain phylogenetic placement (mean *d* = 0.87, 95% CI = −0.15 to 1.89, *k* = 72). The data set was characterised by high total heterogeneity (total *I* ^2^ = 95.93), with 32.45% attributable to phylogenetic history, 29.82% to species‐level differences, 20.54% to study‐level differences, and the remaining 13.11% to observation‐level differences.

**Fig. 2:**
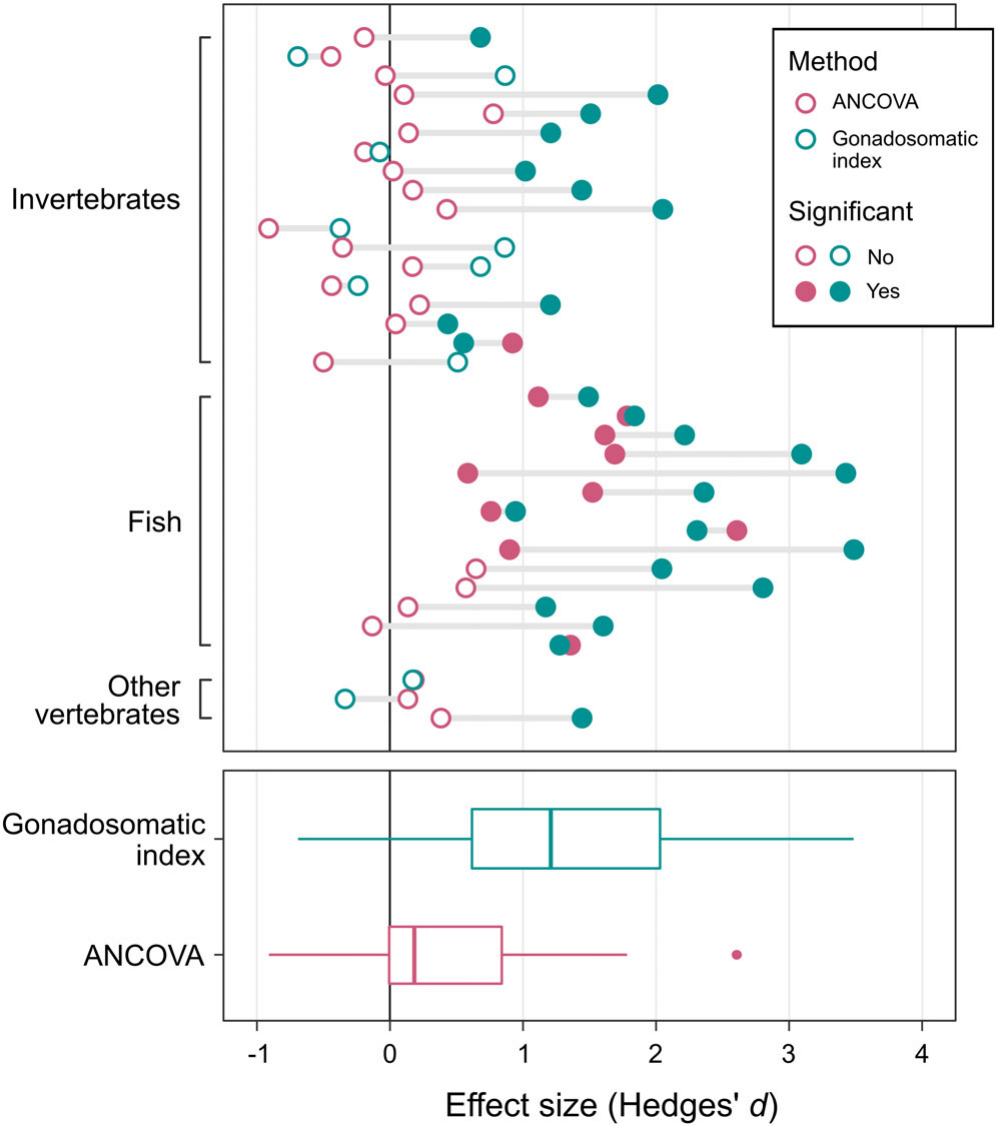
Comparison of two methods for comparing the difference in relative testes size (Hedges' *d*) between male alternative reproductive tactics (ARTs): the gonadosomatic index (blue points) or analysis of covariance (ANCOVA) (red points). Horizontal lines connect effect size estimates derived from the same raw data. Filled and open circles represent cases in which a statistical test (either a *t*‐test or ANCOVA) detected a significant or non‐significant difference respectively in relative testes size between ARTs.

Meta‐regression showed that sneakers have significantly larger testes than non‐sneakers in fish, but there was no difference in invertebrates or other vertebrates (Fig. 1B; Table 2). There was also a significant effect of measurement: sneakers were found to have significantly larger testes than non‐sneakers when using the GSI, but not when using relative testes size (Fig. 1B; Table 2). Importantly, in 29 out of 44 fish studies testes size was measured using the GSI. To test whether the significant difference between tactics in fish could be driven by the inappropriate use of this metric, we used two approaches. First, we estimated the average effect size for fish studies that did not use this metric. After removing GSI effect sizes from the data set, there was no significant difference between sneaker and non‐sneaker males in relative testes size for fish (mean *d* = 1.25, 95% CI = −0.06 to 2.56, *k* = 15; Fig. S6), and no significant difference in mean effect size between the three taxonomic groups (*Q*_M_ = 1.74, *P* = 0.42, *k* = 44, marginal *R* ^2^ = 0.18; Fig. S6). Second, we directly compared effect sizes estimated from the same raw testes allometry data, using both the GSI approach and the recommended ANCOVA approach. For the subset of studies for which raw testes allometry data were available (35 comparisons from 30 species), we found that using the average GSI resulted in a significantly larger difference between male tactics than when using an ANCOVA (paired *t*‐test, *t*_34_ = 6.05, *P* < 0.001; Fig. 2). Importantly, this significant effect remained when only comparing fish species (14 comparisons of 12 species; paired *t*_13_ = 3.95, *P* = 0.002). Further, across all 35 comparisons, using GSI was more likely to result in a statistically significant result (25 of 35 cases, filled circles in Fig. 2) than when using ANCOVA (11 of 35 cases, open circles in Fig. 2).

**Table 2:** Meta‐regression results for all three data sets. Each moderator variable was tested using a separate meta‐regression model. *k* is the number of effect sizes included in each test. The *Q*_M_ statistic tests whether the moderator variable significantly influences the mean effect size. Marginal *R* ^2^ is the amount of variance explained by each moderator. Significant effects are highlighted in grey

Factor	Testes size				Sperm quantity				Sperm traits			
	*k*	*Q*_M_	*P*	Marginal *R* ^2^	*k*	*Q*_M_	*P*	Marginal *R* ^2^	*k*	*Q*_M_	*P*	Marginal *R* ^2^
Taxonomic group	74	16.37	<0.001	0.29	49	0.84	0.66	0.11	128	0.05	0.97	0.002
Mode of fertilisation	74	17.34	<0.001	0.30	49	0.17	0.68	0.001	128	0.23	0.63	0.004
Tactic type	73	0.24	0.63	0.01	45	1.03	0.31	0.02	121	1.38	0.50	0.03
Measurement	71	8.42	0.004	0.12	48	10.23	0.006	0.12	—	—	—	—
Sperm trait	—	—	—	—	—	—	—	—	128	18.52	0.001	0.18
Sperm allocation *vs* expenditure	—	—	—	—	49	1.43	0.23	0.02	—	—	—	—
Sneaker frequency (linear)	62	0.07	0.79	0.001	31	5.77	0.02	0.08	89	1.39	0.24	0.04
Sneaker frequency (quadratic)	62	0.07	0.96	0.001	31	9.1	0.01	0.13	89	1.37	0.5	0.04
Study precision	74	7.54	0.01	0.07	49	0.20	0.66	0.002	128	0.002	0.97	<0.001
Publication year	74	3.38	0.07	0.04	49	4.03	0.04	0.05	128	3.67	0.06	0.06

Meta‐regression showed that sneakers have significantly larger testes than non‐sneakers in species with external fertilisation, but not those with internal fertilisation (Table 2). However, there is an almost total overlap between taxonomic group and fertilisation type in the data set (42 out of 44 effect sizes for fish were from species with external fertilisation), so we cannot separate these two effects (although both factors explain around 30% of the sample variance: Table 2). The difference in testes investment between male ARTs was not influenced significantly by whether tactics were fixed or state‐dependent (Table 2: tactic type). There was no significant linear (slope *β* = −0.25, 95% CI = −2.09 to 1.59) or quadratic relationship between the difference in testes investment between male ARTs and the frequency of sneakers in the population (Table 2). There was a trend for the mean effect size to decrease with study publication year, but not significantly so (*β* = −0.05, 95% CI = −0.10 to 0.003; Table 2). The relationship between effect size and study precision was significantly asymmetric (*β* = −0.28, 95% CI = −0.48 to −0.08; Fig. 1A; Table 2), with a positively skewed distribution. Sample sizes, meta‐analytic means and 95% CIs for each factor level are presented in Table S3.

### (2) Sperm quantity

**Fig. 3:**
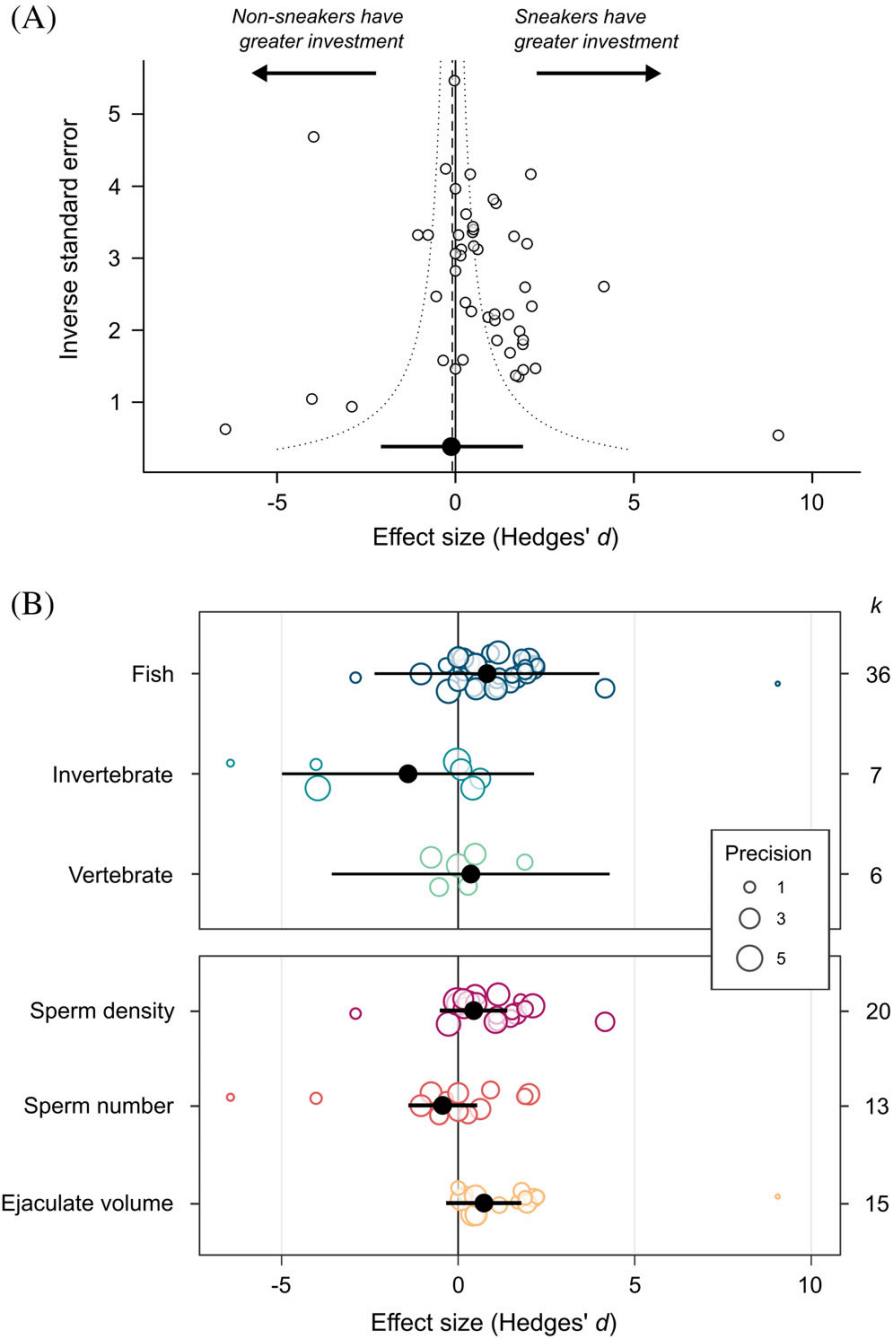
Difference in sperm quantity (Hedges' *d*) between male alternative reproductive tactics in relation to (A) study variance (precision), and (B) taxonomic group (top panel) and quantity measure (bottom panel). In (A), the dashed vertical line represents the meta‐analytic mean, and the dotted lines are the 95% pseudo‐confidence interval. In (B), points are scaled according to study variance (precision). In all panels, black points represent the meta‐analytic mean, and black bars show the 95% confidence interval. *k* = number of effect sizes for each category.

The sperm quantity data set consisted of 49 effect sizes from 43 studies and 32 species. The majority of data came from fish (36 effect sizes, 21 species). We obtained sneaker frequency data for 22 species in this data set. Overall, there was no significant difference in investment in sperm quantity between male ARTs (mean *d* = −0.16, 95% CI = −2.14 to 1.81, *k* = 49; Fig. 3A). This result was the same after removing the four directionless effect sizes (mean *d* = −0.13, 95% CI = −2.19 to 1.94, *k* = 45). The data set was characterised by high total heterogeneity (total *I* ^2^ = 97.04), with 73.68% attributable to phylogenetic history, 10.87% to species‐level differences, 7.03% to study‐level differences, and the remaining 5.45% to observation‐level differences.

**Fig. 4:**
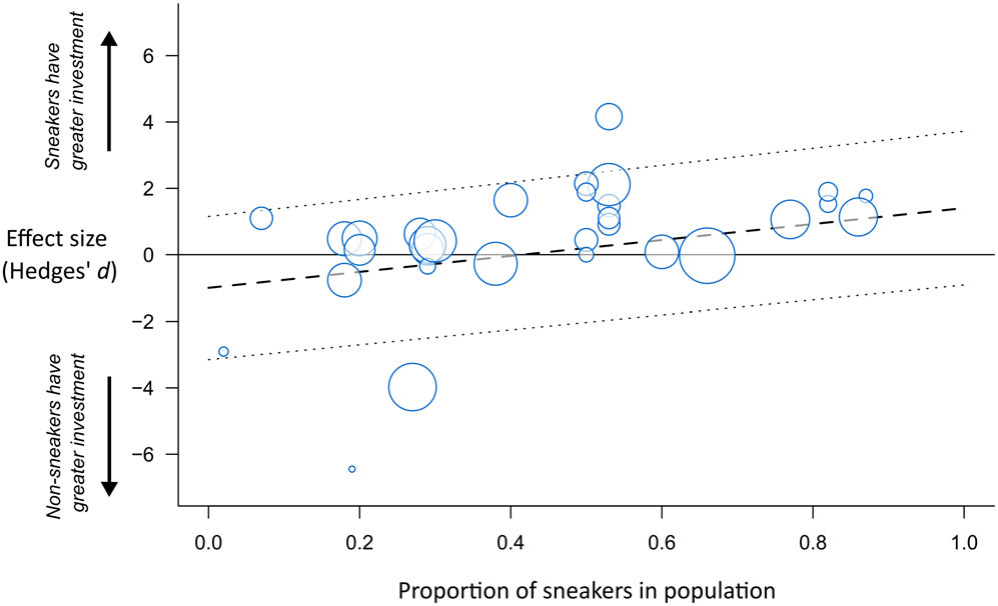
The relationship between the proportion of sneakers in the population and the difference in sperm quantity between male alternative reproductive tactics. Each bubble represents an effect size (*N* = 53), with bubble size scaled to effect size precision (inverse standard error; larger bubbles reflect studies with larger sample sizes). The dashed line shows the predicted line from a meta‐regression including sneaker frequency as a covariate. Dotted lines show the 95% confidence intervals for the predicted line.

The difference in sperm quantity between male ARTs depended on how sperm quantity was measured; sperm density and volume were higher for sneakers, whereas sperm number was higher for non‐sneakers (Fig. 3B; Table 2). However, in no case did the mean estimate differ significantly from zero. The difference in sperm quantity between ARTs was positively related to the proportion of sneakers in the population (*β* = 2.40, 95% CI = 0.44 to 4.36; Table 2; Fig. 4). Adding a quadratic term to the model increased the amount of heterogeneity explained by sneaker frequency (Table 2), but the quadratic term itself did not differ significantly from zero (*z* = −1.76, *P* = 0.08). The difference in sperm quantity between male ARTs was not significantly influenced by taxonomic group (Fig. 3B), fertilisation mode, whether tactics were fixed or state‐dependent (tactic type), or whether sperm expenditure or allocation was examined (Table 2). There was also no effect of study precision (*β* = 0.08, 95% CI = −0.28 to 0.45; Table 2). However, there was a significant negative relationship between effect size and the year in which a study was published (*β* = −0.06, 95% CI = −0.13 to −0.002; Fig. S7; Table 2); this trend appears to be driven by a higher proportion of studies showing negative effects in the last 5 years. Sample sizes, meta‐analytic means and 95% CIs for each factor level are presented in Table S4.

### (3) Sperm traits

**Fig. 5:**
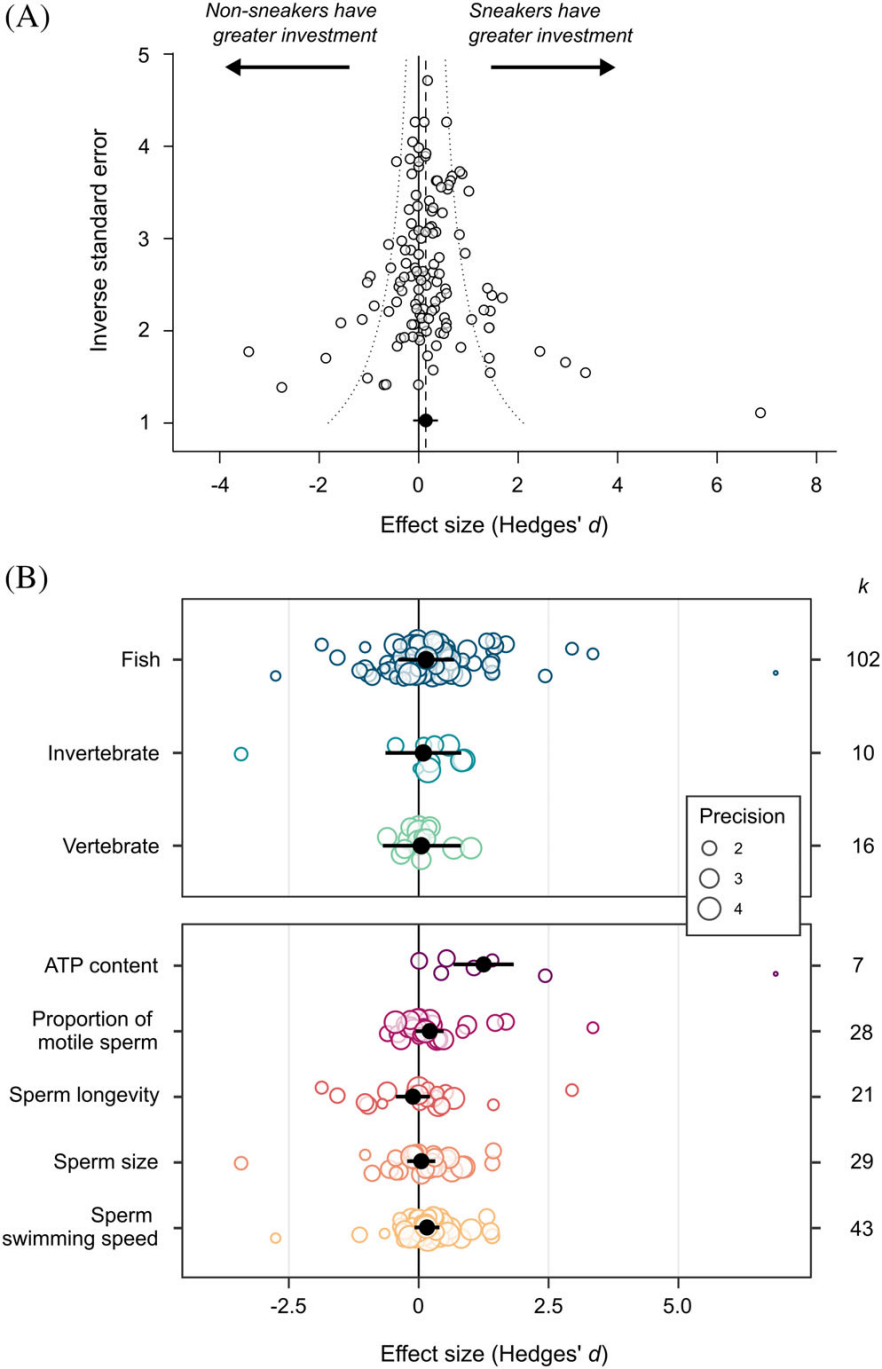
Difference in sperm traits (Hedges' *d*) between male alternative reproductive tactics in relation to (A) study variance (precision), and (B) taxonomic group (top panel) and sperm trait (bottom panel). In (A), the dashed vertical line represents the meta‐analytic mean, and the dotted lines are the 95% pseudo‐confidence interval. In (B), points are scaled according to study variance (precision). In all panels, black points represent the meta‐analytic mean, and black bars show the 95% confidence interval. *k* = number of effect sizes for each category.

The sperm traits data set consisted of 128 effect sizes from 55 studies and 33 species. The majority of data came from fish (102 effect sizes, 22 species). We obtained sneaker frequency data for 23 species in this data set. Overall, there was no significant difference in sperm traits between male ARTs (mean *d* = 0.14, 95% CI = −0.05 to 0.33, *k* = 128; Fig. 5A). This result was the same after removing the 10 directionless effect sizes (mean *d* = 0.15, 95% CI = −0.04 to 0.35, *k* = 118), and after incorporating a variance matrix to account for potential non‐independence of sperm traits measured on the same males (Table S5). The data set was characterised by high total heterogeneity (total *I* ^2^ = 74.8%), with 0.9% attributable to phylogenetic history, 17.1% to species‐level differences, 8.72% to study‐level differences, and the remaining 48.1% to observation‐level differences.

The difference in sperm traits between male ARTs differed according to which sperm trait was measured (Table 2). However, only ATP content had an estimate that differed significantly from zero (Fig. 5B). The difference in sperm traits between male ARTs was not significantly influenced by taxonomic group (Fig. 5B), mode of fertilisation, or tactic type (Table 2). There was no significant linear (*β* = −0.32, 95% CI = −0.86 to 0.21) or quadratic relationship between the difference in sperm traits between male ARTs and the frequency of sneakers in the population (Table 2). There was also no significant relationship between the difference in sperm traits between male ARTs and study precision (*β* = −0.004, 95% CI = −0.20 to 0.19; Table 2). There was a marginally non‐significant trend for the mean effect size to decrease with study publication year (*β* = −0.02, 95% CI = −0.05 to 0.0006; Table 2). Sample sizes, meta‐analytic means and 95% CIs for each factor level are presented in Table S5.

## IV DISCUSSION

We systematically searched the literature for studies comparing ejaculate investment and sperm traits between males using different types of ARTs. We found data from 92 studies and 67 species; more than double the 29 species surveyed by Kustra & Alonzo (2020). Despite this larger data set, our quantitative results broadly matched their qualitative results. We found that, after controlling for body size, male fish (but not any other taxonomic groups) using tactics that elevate sperm competition risk, or that had a reduced investment in traits that increase mating success, had significantly larger testes than males using other alternative tactics. However, this pattern disappears when we restrict the analysis to those studies that do not use the GSI as a measure of testes investment. Males exhibiting different ARTs did not differ significantly in sperm number (either sperm allocation or expenditure), nor in other sperm traits, with the exception of sperm ATP content in fish. We failed to detect the predicted quadratic relationship between sneaker frequency and the difference in post‐mating investment between ARTs in any of the three data sets. However, we did detect a significant positive linear relationship between sneaker frequency and the difference in sperm quantity between ARTs, thus showing that the abundance of sneakers does influence the average ejaculate investment of males exhibiting ARTs to some extent. Finally, contrary to our predictions, differences in testes size, sperm number or sperm traits between male ARTs were unaffected by the extent to which tactics were flexible.

### (1) Appraising the evidence

In fishes, males exhibiting tactics associated with an increased risk of sperm competition, or a reduced investment in traits that increase mating success, had relatively larger testes than males exhibiting alternative tactics. This result supports predictions based on sperm competition theory (Parker, 1990a,b; Gage *et al*., 1995; Ball & Parker, 2003; Parker & Pizzari, 2010). Why is this relationship present in fish but not in any other taxonomic group? We suggest three potential explanations. First, more data were available for fish than for other taxonomic groups, increasing our statistical power (Kustra & Alonzo, 2020). Second, almost all (26 of 28) of the fish species in the testes size data set exhibit external fertilisation, whereas the vast majority (24 of 25) of the remaining species exhibit internal fertilisation. This pattern might therefore be explained by differences in fertilisation mode, given that: (*i*) sperm limitation is likely to be more important in external fertilisers; and (*ii*) strong sperm precedence or cryptic female choice in internal fertilisers is expected to weaken the relationship between sperm number and fertilisation (Fitzpatrick, 2020). However, we found no effect of fertilisation mode in the sperm quantity or sperm traits data sets. Further testing of this relationship is difficult without more data on internally fertilising fish species showing ARTs.

Third, and most importantly, the use of the GSI as a measure of testes size is widespread in studies of fish, but rare in other taxa. In the testes size data set, 29 of 44 fish effect sizes used the GSI approach, whereas only 1 of 30 of the non‐fish effect sizes did. As discussed in Section (2), the GSI is an unsuitable metric to use when comparing male tactics, because it only controls properly for body size when the relationship between testes size and body size is isometric (Tomkins & Simmons, 2002). When the slope of the relationship between testes size and body size is less than 1, the difference in testes investment between large and small male morphs is overestimated. Instead, the use of an ANCOVA is recommended, which directly accounts for positive or negative allometry, as well as differences in allometry between male morphs (Tomkins & Simmons, 2002). We provide two forms of evidence that the significant difference in testes investment seen for fish is driven by the use of this inappropriate metric. First, the effect disappears when studies using the GSI to measure testes investment are excluded. Second, re‐analysis of raw testes allometry data (35 comparisons, 30 species) showed that the GSI approach resulted in a significantly larger effect size than the ANCOVA approach, both for the full data set and when only considering fish. We believe this is the strongest evidence yet that GSI is an inappropriate method to compare testes investment between male ARTs.

We found no evidence for differences in sperm quantity or sperm traits between male ARTs that differ in sperm competition risk. The only exception was sperm ATP content in fish. Across five species of fish, sneaker male sperm contained more ATP per cell than non‐sneaker male sperm. Intraspecific studies have shown a positive relationship between ATP content and sperm motility (e.g. Christen, Gatti & Billard, 1987; Perchec *et al*., 1995; Burness *et al*., 2004). However, the ATP content of a sperm cell depends on the balance between production before and after ejaculation (either through respiration or glucose or lipid catabolism; Werner & Simmons, 2008), and consumption during cellular maintenance and motility (Tourmente *et al*., 2019). This means that high cell ATP content could potentially reflect high initial stores, high production after ejaculation, low consumption, or a combination of all three (e.g. Christen *et al*., 1987). All of the effect sizes in our data set reflect stored ATP levels, as ATP content was measured in stripped (not ejaculated) sperm, immediately after sampling, and before activation by contact with fresh water or sea water. It is therefore unclear whether this difference between male tactics also exists for ATP production or consumption. Nevertheless, we suggest this result should be interpreted with caution, for two reasons. First, it is derived from only seven effect sizes, from six studies (Table S5). Second, sperm ATP content is assumed to improve fertilisation success by increasing sperm swimming speed, motility or longevity (or all three). However, none of these three traits differed between male tactics in our data set, even though we obtained larger sample sizes than those for ATP content. Our ability to detect a significant difference in sperm traits between ARTs could have been reduced because we combined estimates from multiple sperm traits which may exhibit functional or resource‐allocation trade‐offs (Snook, 2005). However, widespread trade‐offs in the same direction would be revealed in our analysis *via* differences in the average sign of the effect size for different sperm traits. For example, a speed–longevity trade‐off could result in a positive effect size for sperm swimming speed and a negative effect size for sperm longevity. However, when considering each sperm trait separately, only sperm ATP content differed significantly between male tactics (Table S5), which suggests that such trade‐offs do not act in the same direction across species, at least in relation to differences in ART. Indeed, such trade‐offs are also not apparent when comparing multiple sperm traits between ARTs within the same species (Kustra & Alonzo, 2020). This suggests either that such trade‐offs do not typically constrain the evolution of sperm traits across the animal kingdom, or that species can solve any trade‐offs in multiple ways.

Theory predicts that the difference in post‐mating investment between guarders and sneakers should be greatest when sneakers are at an intermediate frequency in the population (Parker, 1990b; Gage *et al*., 1995). We failed to confirm this prediction: there was no significant quadratic relationship between sneaker frequency and the difference in post‐mating investment between ARTs in any of the three data sets. However, there was a significantly positive linear relationship between sneaker frequency and effect size for the sperm quantity data set, even though the average difference between ARTs was close to zero. Such a linear relationship could arise due to a lack of data at high sneaker frequencies, which reduces our power to detect the predicted decrease in the disparity between ARTs in this region. We thus consider this to be tentative evidence showing that the abundance of sneakers does indeed influence the average ejaculate investment of males exhibiting alternative tactics to some extent. We may have failed to find a relationship between sneaker frequency and the difference in post‐mating investment between ARTs for the testes size and sperm traits data sets because of data limitations. For example, we were typically only able to obtain an estimate of sneaker frequency from a single population for each species, even though for some species we had post‐mating trait data from more than one population. Therefore, there may be important among‐population variation in sneaker frequency that we could not account for. It is also important to note that the average frequency of sneakers in the population is related to, but not identical to, the average frequency of *sneaking* per mating event. The difference between sneaker and sneaking frequency can often be large. For example, in the cichlid *Lamprologus callipterus* dwarf (sneaker) males may comprise around half of the population, but were found to participate in only 5% of observed spawning events (Wirtz Ocana *et al*., 2014). Additionally, the frequency of sneaking is likely to be very variable across the breeding season and depending on the immediate social and abiotic environment. Such variability may be relevant for sperm traits which can be varied rapidly in response to immediate social cues, but less relevant for traits such as testes size which change over evolutionary time. However, this does not mean that sneaker frequency is an irrelevant metric when considering post‐mating traits. This is because sneaker frequency tells us what the evolutionarily stable frequency of each male tactic is, which influences the *average* sperm competition risk across all contexts and individuals. If this average risk differs between ARTs, then it will influence the optimal investment into sperm and ejaculate traits irrespective of spatial or temporal variation in sneaking frequency. Importantly, such a stable frequency exists for both fixed tactics [in which the relative reproductive success of male ARTs is stabilised at equilibrium by negative frequency‐dependent selection (Gross, 1991; Shuster & Wade, 1991)] and state‐dependent tactics; in the latter case, the frequency of high‐quality ‘dominant’ males in the population will influence the threshold at which poor‐condition individuals switch to an alternative tactic (e.g. Tomkins & Brown, 2004).

All three data sets were characterised by very high heterogeneity. While high heterogeneity is commonly seen in ecological meta‐analyses (Senior *et al*., 2016), it does reduce the power of the analysis to detect small effects due to putative moderators, if other sources of variation cannot readily be identified and accounted for. Partitioning of heterogeneity suggested that the proportion of variation explained by species‐level and phylogenetic differences combined was high for both the testes size data set (62%) and the sperm quantity data set (85%). This suggests that both of these traits evolve slowly, possibly because of constraints on testes function. By contrast, for the sperm traits data set only 18% of heterogeneity could be attributed to phylogenetic or species‐level differences, suggesting fewer constraints on their evolution. Notably, the proportion of variance explained by any of the nine tested moderator variables was small for all three data sets (with the exception of taxonomic group and mode of fertilisation for the testes size data set). Therefore, much of the effect size heterogeneity remains unexplained, especially for the sperm traits data set. Several factors could explain this heterogeneity, including complex changes in the immediate social environment (e.g. local variation in the number and types of rival males present during spawning), other species‐specific selection pressures on male post‐mating traits [e.g. *L. callipterus* sneaker males face a higher sperm competition risk but occupy a favoured role during spawning (Schutz *et al*., 2010; Taborsky *et al*., 2018); see Section (3)], and functional trade‐offs between sperm traits [e.g. a trade‐off between swimming speed and sperm longevity (Levitan, 2000); see Section (3)].

### (2) Publication bias

We detected some evidence for publication bias in the three data sets. All three data sets showed a decrease in the mean effect size over time, although only significantly so for sperm quantity. Hence, studies showing no difference in post‐mating traits between male ARTs, or a difference in the opposite direction to that typically predicted, are now published more often than in the 1990s. This could be for a variety of reasons, including an increase in sample size or improved methodological rigour over time, changes in editorial policy or in the types of study systems being investigated, or the fact that early theoretical investigations (e.g. Parker, 1990a,b; Gage *et al*., 1995) were influential and led to a genuine publication bias against non‐confirmatory results. The funnel plot for testes sizes was significantly asymmetric, with a positively‐skewed distribution. This pattern could arise if studies reporting a negative effect size are less likely to be published. However, our analysis indicated that the testes size data set was significantly heterogeneous in relation to taxonomic group, fertilisation mode and measurement type. We therefore suggest that the asymmetry is driven by true heterogeneity in the data set, rather than biased publication practices (Nakagawa & Santos, 2012).

### (3) Explaining the incongruence between theory and data

Taken together, these results suggest that the current empirical evidence that male ARTs differ consistently in their investment into sperm and ejaculates is very weak. This is surprising, given that almost all theoretical models predict that sneaker males should invest more than non‐sneaker males into post‐mating traits (Parker, 1990a,b; Gage *et al*., 1995; Ball & Parker, 2003). We have several potential explanations for the incongruence between theory and empirical data. First, males exhibiting ARTs may not differ significantly in sperm competition risk. One reason for this would be if sneakers typically make up a high proportion of males in the population (Parker, 1990b; Gage *et al*., 1995; Simmons *et al*., 2007). We obtained these data for 53 species across all three data sets. Across these 53 species, sneaker frequency ranged from 2% of males in the cichlid *Amatliana siquia* (Clotfelter *et al*., 2017), to 87% of males in the dusky frillgoby *Bathygobius fuscus* (Takegaki, Kaneko & Matsumoto, 2012), with an average of 39% (Fig. S5). Importantly, non‐sneakers outnumber sneakers by 2:1 or more in only 23 of the 53 species, and in fact sneakers outnumber non‐sneakers in 18 of the remaining 30 species. Therefore, sneaker males are certainly not rare for the majority of species in our sample, so that non‐sneakers may typically face a similar sperm competition risk to sneakers (assuming sneaker frequency is a reasonable proxy for the frequency of breeding events that involve sperm competition; but see Wirtz Ocana *et al*., 2014). Second, males often face multiple selection pressures in relation to sperm and ejaculate investment. For example, in the cichlid fish *L. callipterus*, dwarf (sneaker) males attempt to steal fertilisations from larger, nesting males (Schutz *et al*., 2010; Taborsky *et al*., 2018). However, females spawn in empty shells collected by nesting males, and their small size means that sneaker males can enter these shells during spawning and ejaculate much closer to the eggs than can nesting males (Schutz *et al*., 2010; Taborsky *et al*., 2018). Thus, while nesting males generally face lower sperm competition than sneaking males, they also occupy a disfavoured role, and could benefit from investing more into sperm and ejaculate traits to compensate. This example illustrates how multiple factors may act simultaneously to influence sperm investment of different ARTs in complex ways.

Sperm competition models are also simplistic in three key ways. First, models assume that fertilisation is the result of a ‘fair raffle’, whereby a male's chance of fertilising a female's eggs is directly proportional to how many sperm he produces (Parker, 1990a,b; Gage *et al*., 1995; Ball & Parker, 2003). This assumption may be met in broadcast‐spawning external fertilisers, but such species rarely show ARTs (and no examples are present in this analysis). By contrast, in many other external fertilisers, a male's proximity to a female during gamete release may be much more important than how many sperm he produces (Taborsky *et al*., 2018), and in internal fertilisers first‐ or last‐male sperm precedence or cryptic female choice (a ‘loaded raffle’) will act to obscure the relationship between sperm number and fertilisation success (Simmons, 2001). Second, models do not consider functional trade‐offs between post‐mating traits (Kustra & Alonzo, 2020) which could limit the ability of ejaculate or sperm traits to evolve independently of each other (Snook, 2005; Simmons & Fitzpatrick, 2012). Third, models typically assume that males exhibiting different ARTs have the same overall energy budget, which they divide differentially between pre‐ and post‐mating traits (Kustra & Alonzo, 2020). However, in species with state‐dependent ARTs sneaker males will be in poorer condition than non‐sneaker males, and hence less able to afford to increase their absolute investment into sperm or ejaculate traits. The fact that ejaculate and sperm traits may also be influenced by individual condition or diet (Macartney *et al*., 2019) suggests that sneaker males may often be unable to produce larger ejaculates or higher‐quality sperm because of energetic limitations. Males exhibiting ARTs may also differ in resource allocation even when the choice of tactic is not condition dependent. For example, at certain points in the breeding season guarding males may have few resources to invest into ejaculates because of the conflicting demands of territory defence, female courtship and brood care (Taborsky, 2008).

It has also been questioned whether the traits commonly measured in empirical studies are appropriate proxies for post‐mating investment. For example, as discussed above, GSI has been criticised as an inappropriate measure of size‐corrected investment in testes tissue (Tomkins & Simmons, 2002). Sperm competition risk is not the only factor that influences ejaculate size or sperm production; large testes may also be important for males with high mating rates independent of levels of sperm competition (Vahed & Parker, 2012) or in species in which females lay large clutches (Emerson, 1997). Additionally, the relationship between sperm traits and fertilisation ability is complex (Snook, 2005; Simmons & Fitzpatrick, 2012), and predictions are often based on verbal arguments with dubious assumptions. For example, the general assumption that longer sperm are better swimmers is likely to be unfounded, especially for internal fertilisers (Humphries, Evans & Simmons, 2008). It may be more appropriate in future to focus on sperm traits that have stronger causal links to sperm performance, such as the ratio of flagellum length to head length (Humphries *et al*., 2008), or sperm ATP content (Tourmente *et al*., 2019).

There are also other ejaculate components that we did not consider here but which may play an important role in mediating male fertilisation success (Kustra & Alonzo, 2020). For example, studies of fish with male ARTs have shown that both the amount (Poli *et al*., 2018) and composition of the male seminal fluid differs between tactics (Gombar *et al*., 2017). Further, seminal fluid may improve the competitiveness of sperm from the same males (Locatello *et al*., 2013; Bartlett *et al*., 2017; Poli *et al*., 2018; Gasparini, Pilastro & Evans, 2020), or even reduce the competitiveness of sperm from males exhibiting the alternative tactic (Locatello *et al*., 2013; Lewis & Pitcher, 2017b). This latter observation raises the possibility that sneaker and guarder males could be engaged in a molecular ‘arms race’, with sneaker males evolving seminal fluid components that impair guarder sperm competitiveness, and guarders evolving traits that resist the effect of these components. Nevertheless, differences in seminal fluid between male ARTs have been investigated in only three fish species. Until we have more data, we cannot rule out the possibility that, when compared to non‐sneaker males, sneaker males consistently produce more seminal fluid per mating, or produce non‐sperm components of the ejaculate that are more competitive. Another factor which has been mostly ignored is cryptic female choice, which occurs in both internal and external fertilisers and has the potential to alter the relative competitiveness of sperm from different tactics (Simmons, 2001; Fitzpatrick, 2020). For example, in the ocellated wrasse *Symphodus ocellatus* female ovarian fluid increases sperm swimming speed, and this likely enhances the competitiveness of dominant males, who produce fewer, faster sperm than sneaker males (Alonzo, Stiver & Marsh‐Rollo, 2016).

### (4) Future directions

In summary, our meta‐analyses show that the current evidence for consistent differential investment into post‐mating traits by males exhibiting different ARTs is weak, especially in relation to sperm quantity and individual sperm traits. However, all three data sets were characterised by high heterogeneity, well beyond that attributable to sampling error alone, which remains mostly unexplained. It remains unclear if the incongruence between data and theory is due to theory not taking real‐world complexity into account, empirical studies that focus on the wrong post‐mating traits, or both. However, there is clearly a need to reassess the validity of the assumptions underlying mathematical models of sperm competition. For example, the assumption that fertilisation follows a fair raffle is likely to be unrealistic for most species (Simmons, 2001). If such assumptions do not apply widely, it does not mean that a model is incorrect; rather that only species that match these assumptions are appropriate test subjects. Further, it may be naïve to expect to see the same general patterns across divergent taxa given how much species vary, even within the same genus. While the disparate species represented in our meta‐analysis do indeed exhibit similar ARTs, there are many important biological and ecological differences among species (for example in their intra‐ and inter‐sexual interactions, the importance of different sperm traits for determining male fertilisation success, or the mechanisms of sperm utilisation by females) which could obscure any general patterns. In light of these points, we have several clear recommendations for researchers. First, the GSI should not be used to compare gonadal investment between male tactics. This is not a new recommendation, but we hope that by expanding the original comparison by Tomkins & Simmons (2002) from 5 to 30 species, we provide very strong evidence in support of abandoning the GSI. Second, we need more empirical data linking sperm traits to fertilisation success in target species. As it is, we are in danger of measuring sperm quality using traits that do not directly influence sperm competitiveness (Snook, 2005; Simmons & Fitzpatrick, 2012). We should also not assume that the post‐mating traits that partially determine male fertilisation success in one or a few species will do so in all species or different types of ART. Finally, we need new theory which takes into account complexities driven by the social environment, energetic constraints and male physiology, sperm function, and functional trade‐offs between post‐mating traits (Kustra & Alonzo, 2020).

## V CONCLUSIONS

(1) We performed three meta‐analyses examining how testes size, sperm number and sperm traits differ between males exhibiting ARTs that face either a high or a low sperm competition risk, or have high or low investment in traits that increase mating success.

(2) Male fish exhibiting ARTs facing a high sperm competition risk had significantly larger testes after controlling for body size than those exhibiting tactics facing a low sperm competition risk. However, we suggest this difference is driven by the widespread use of GSI as a measure of testes investment in fish, which overestimates the difference in testes investment between male tactics when the relationship between testes size and body size is not isometric.

(3) There was no significant difference in sperm quantity between males exhibiting different ARTs, regardless of whether it was measured in the testes or following ejaculation.

(4) There was no significant difference in sperm traits between males exhibiting different ARTs, except for sperm ATP content in fish.

(5) The difference in post‐mating investment between male ARTs was not influenced by taxonomic group or by the extent to which tactics were flexible. However, the difference in sperm quantity between ARTs increased as sneakers became more common in the population. The difference in testes size between male ARTs was greater for external than internal fertilisers.

(6) Overall, there is little evidence that male ARTs differ substantially in investment into sperm and ejaculates. The incongruence between theoretical and empirical results could be explained if (*i*) theoretical models fail to account for differences in overall resource levels between males exhibiting different ARTs or fundamental trade‐offs between investment into different ejaculate and sperm traits, and (*ii*) studies often use sperm or ejaculate traits that do not reflect overall post‐mating investment or relate to fertilisation success.

(7) We recommend that future studies: (*i*) cease using the GSI to quantify gonadal investment; (*ii*) seek empirical data linking specific sperm traits to fertilisation success in a range of species; (*iii*) compare non‐sperm components of the ejaculate between male ARTs; and (*iv*) develop theoretical models that take into account the presence of multiple selection pressures acting on male post‐mating investment, variable patterns of sperm precedence, differences in energy budgets between males exhibiting ARTs, and functional trade‐offs between sperm traits.

## VI ACKNOWLEDGEMENTS, AUTHOR CONTRIBUTIONS AND DATA ACCESSIBILITY

We thank Jasmine Loveland, Yuya Makiguchi, Jessica Miller, and Bryan Neff for sending data, and Michael Taborsky and an anonymous reviewer for comments which greatly improved the manuscript. This work was supported by a Leverhulme Trust Early Career Fellowship to L.R.D. (ECF‐2018‐427).

*Author contributions*: L.R.D. developed the methods, screened studies, extracted data, performed meta‐analysis, and wrote the first draft of the paper. M.J.A.S. developed the methods and screened studies. M.D.J. and L.W.S. conceived the study, developed the methods, and contributed to writing.

*Data accessibility*: All data and code used in the analysis are available from Figshare (10.6084/m9.figshare.19174604).

## Supporting information


**Appendix S1.** PRISMA‐EcoEvo checklist.

**Fig. S1.** PRISMA diagram summarising the literature search and study screening processes.
**Table S1.** Methods for calculating the standardised mean difference (Hedges' *d*) and the location of the data collected.
**Fig. S2.** Phylogenetic tree for the 53 species in the testis size data set.
**Fig. S3.** Phylogenetic tree for the 32 species in the sperm quantity data set.
**Fig. S4.** Phylogenetic tree for the 33 species in the sperm traits data set.
**Table S2.** Sources used for the sneaker frequency data.
**Fig. S5.** Histogram showing the distribution of sneaker frequency across 54 species.
**Fig. S6.** Differences in testes size (Hedges' *d*) between male alternative reproductive tactics (ARTs) in relation to taxonomic group, after removing studies using the gonadosomatic index.
**Table S3.** Mean effect size estimates (Hedges' *d*), 95% confidence intervals, and sample sizes for the testis size data set.
**Fig. S7.** Relationship between effect size (Hedges' *d*) and publication year for the sperm quantity data set.
**Table S4.** Mean effect size estimates (Hedges' *d*), 95% confidence intervals, and sample sizes for the sperm quantity data set.
**Table S5.** Mean effect size estimates (Hedges' *d*), 95% confidence intervals, and sample sizes for the sperm traits data set.
