# Supplementary material for: Male alternative reproductive tactics and sperm competition: a meta‐analysis
Source: Biol Rev Camb Philos Soc. 2022 Feb 28;97(4):1365–88. doi: 10.1111/brv.12846 (PMC9541908; doi:10.1111/brv.12846)
Supplement: Supplementary file 1 — Appendix S1. PRISMA‐EcoEvo checklist. [file BRV-97-1365-s001.docx]

**Appendix S1: PRISMA-EcoEvo checklist**

Male alternative reproductive tactics and sperm competition: a meta-analysis

Liam R. Dougherty^1*^, Michael J. A. Skirrow^2^, Michael D. Jennions^2^, and Leigh W. Simmons^3^

^1^*Department of Evolution, Ecology and Behaviour; University of Liverpool; Crown Street; Liverpool; L69 7RB; UK*

^2^*Evolution, Ecology and Genetics, Research School of Biology, The Australian National University, Canberra, ACT, 0200, Australia*

^3^*Centre for Evolutionary Biology, School of Biological Sciences, The University of Western Australia, Crawley, WA, 6009, Australia*

^*^E-mail: liam.dougherty@liverpool.ac.uk Tel: (+44) 0151 795 7771

The PRISMA-EcoEvo extension was published in 2021 (O’Dea *et al*., 2021). It consists of a 27-item checklist and guidance for reporting systematic reviews and meta-analyses of primary research in ecology and evolutionary biology. Within each item, sub-items are given a percentage score (calculated using the Shiny app: <https://prisma-ecoevo.shinyapps.io/checklist/>). Higher item scores thus indicate that a higher proportion of sub-items are reported in the manuscript.

| **Checklist item** | **Item score** | **Sub-item number** | **Sub-item** | **Reported by authors?** | **Notes** |
| --- | --- | --- | --- | --- | --- |
| **Title and abstract** | **100%** | 1.1 | Identify the review as a systematic review, meta-analysis, or both | Yes | Page 2 |
|  |  | 1.2 | Summarise the aims and scope of the review | Yes | Page 2 |
|  |  | 1.3 | Describe the data set | Yes | Page 2 |
|  |  | 1.4 | State the results of the primary outcome | Yes | Page 2-3 |
|  |  | 1.5 | State conclusions | Yes | Page 3 |
|  |  | 1.6 | State limitations | Yes | Page 2-3 |
| **Aims and questions** | **80%** | 2.1 | Provide a rationale for the review | Yes | Page 9 |
|  |  | 2.2 | Reference any previous reviews or meta-analyses on the topic | Yes | Page 9 |
|  |  | 2.3 | State the aims and scope of the review (including its generality) | Yes | Page 14 |
|  |  | 2.4 | State the primary questions the review addresses (e.g. which moderators were tested) | Yes | Pages 14-15 |
|  |  | 2.5 | Describe whether effect sizes were derived from experimental and/or observational comparisons | No | n/a |
| **Review registration** | **0%** | 3.1 | Register review aims, hypotheses (if applicable), and methods in a time-stamped and publicly accessible archive and provide a link to the registration in the methods section of the manuscript. Ideally registration occurs before the search, but it can be done at any stage before data analysis. | No | n/a |
|  |  | 3.2 | Describe deviations from the registered aims and methods | No | n/a |
|  |  | 3.3 | Justify deviations from the registered aims and methods | No | n/a |
| **Eligibility criteria** | **100%** | 4.1 | Report the specific criteria used for including or excluding studies when screening titles and/or abstracts, and full texts, according to the aims of the systematic review (e.g. study design, taxa, data availability) | Yes | Pages 17-21 |
|  |  | 4.2 | Justify criteria, if necessary (i.e. not obvious from aims and scope) | Yes | Pages 17-21 |
| **Finding studies** | **100%** | 5.1 | Define the type of search (e.g. comprehensive search, representative sample) | Yes | Pages 15-17 |
|  |  | 5.2 | State what sources of information were sought (e.g. published and unpublished studies, personal communications) | Yes | Page 15 |
|  |  | 5.3 | Include, for each database searched, the exact search strings used, with keyword combinations and Boolean operators | Yes | Pages 15-16 |
|  |  | 5.4 | Provide enough information to repeat the equivalent search (if possible), including the timespan covered (start and end dates) | Yes | Page 15 |
| **Study selection** | **100%** | 6.1 | Describe how studies were selected for inclusion at each stage of the screening process (e.g. use of decision trees, screening software) | Yes | Page 17 |
|  |  | 6.2 | Report the number of people involved and how they contributed (e.g. independent parallel screening) | Yes | Page 17 |
| **Data collection process** | **67%** | 7.1 | Describe where in the reports data were collected from (e.g. text or figures) | Yes | Page 21-22 |
|  |  | 7.2 | Describe how data were collected (e.g. software used to digitize figures, external data sources) | Yes | Page 22 |
|  |  | 7.3 | Describe moderator variables that were constructed from collected data (e.g. number of generations calculated from years and average generation time) | No | n/a |
|  |  | 7.4 | Report how missing or ambiguous information was dealt with during data collection (e.g. authors of original studies were contacted for missing descriptive statistics, and/or effect sizes were calculated from test statistics) | Yes | Page 22 |
|  |  | 7.5 | Report who collected data | Yes | Page 22 |
|  |  | 7.6 | State the number of extractions that were checked for accuracy by co-authors | No | n/a |
| **Data items** | **100%** | 8.1 | Describe the key data sought from each study | Yes | Page 21-22 |
|  |  | 8.2 | Describe items that do not appear in the main results, or which could not be extracted due to insufficient information | Yes | Page 21-22 |
|  |  | 8.3 | Describe main assumptions or simplifications that were made (e.g. categorising both 'length' and 'mass' as 'morphology') | Yes | Pages 17-21 |
|  |  | 8.4 | Describe the type of replication unit (e.g. individuals, broods, study sites) | Yes | Page 27 |
| **Assessment of individual study quality** | **0%** | 9.1 | Describe whether the quality of studies included in the systematic review or meta-analysis was assessed (e.g. blinded data collection, reporting quality, experimental versus observational) | No | n/a |
|  |  | 9.2 | Describe how information about study quality was incorporated into analyses (e.g. meta-regression and/or sensitivity analysis) | No | n/a |
| **Effect size measures** | **100%** | 10.1 | Describe effect size(s) used | Yes | Page 21 |
|  |  | 10.2 | Provide a reference to the equation of each calculated effect size (e.g. standardised mean difference, log response ratio) and (if applicable) its sampling variance | Yes | Pages 21-22 |
|  |  | 10.3 | If no reference exists, derive the equations for each effect size and state the assumed sampling distribution(s) | No | n/a |
| **Missing data** | **0%** | 11.1 | Describe any steps taken to deal with missing data during analysis (e.g. imputation, complete case, subset analysis) | No | n/a |
|  |  | 11.2 | Justify the decisions made to deal with missing data | No | n/a |
| **Meta-analytic model description** | **100%** | 12.1 | Describe the models used for synthesis of effect sizes | Yes | Pages 27-28 |
|  |  | 12.2 | The most common approach in ecology and evolution will be a random-effects model, often with a hierarchical/multilevel structure. If other types of models are chosen (e.g. common/fixed effects model, unweighted model), provide justification for this choice | No | n/a |
| **Software** | **100%** | 13.1 | Describe the statistical platform used for inference (e.g. R) | Yes | Page 27 |
|  |  | 13.2 | Describe the packages used to run models | Yes | Page 27 |
|  |  | 13.3 | Describe the functions used to run models | Yes | Page 27 |
|  |  | 13.4 | Describe any arguments that differed from the default settings | No | n/a |
|  |  | 13.5 | Describe the version numbers of all software used | Yes | Page 27 |
| **Non-independence** | **100%** | 14.1 | Describe the types of non-independence encountered (e.g. phylogenetic, spatial, multiple measurements over time) | Yes | Pages 27-28 |
|  |  | 14.2 | Describe how non-independence has been handled | Yes | Pages 27-28 |
|  |  | 14.3 | Justify decisions made | Yes | Pages 27-28 |
| **Meta-regression and model selection** | **50%** | 15.1 | Provide a rationale for the inclusion of moderators (covariates) that were evaluated in meta-regression models | Yes | Page 28 |
|  |  | 15.2 | Justify the number of parameters estimated in models, in relation to the number of effect sizes and studies (e.g. interaction terms were not included due to insufficient sample sizes) | No | n/a |
|  |  | 15.3 | Describe any process of model selection | No | n/a |
| **Publication bias and sensitivity analysis** | **100%** | 16.1 | Describe assessments of the risk of bias due to missing results (e.g. publication, time-lag, and taxonomic biases) | Yes | Page 29 |
|  |  | 16.2 | Describe any steps taken to investigate the effects of such biases (if present) | Yes | Page 29 |
|  |  | 16.3 | Describe any other analyses of robustness of the results, e.g. due to effect size choice, weighting or analytical model assumptions, inclusion or exclusion of subsets of the data, or the inclusion of alternative moderator variables in meta-regressions | Yes | Page 28 |
| **Clarification of post hoc analyses** | **0%** | 17.1 | When hypotheses were formulated after data analysis, this should be acknowledged. | No | n/a |
| **Metadata, data, and code** | **100%** | 18.1 | Share metadata (i.e. data descriptions) | Yes | 10.6084/m9.figshare.19174604 |
|  |  | 18.2 | Share data required to reproduce the results presented in the manuscript | Yes | 10.6084/m9.figshare.19174604 |
|  |  | 18.3 | Share additional data, including information that was not presented in the manuscript (e.g. raw data used to calculate effect sizes, descriptions of where data were located in papers) | Yes | 10.6084/m9.figshare.19174604 |
|  |  | 18.4 | Share analysis scripts (or, if a software package with graphical user interface (GUI) was used, then describe full model specification and fully specify choices) | Yes | 10.6084/m9.figshare.19174604 |
| **Results of study selection process** | **100%** | 19.1 | Report the number of studies screened | Yes | Figure 1 |
|  |  | 19.2 | Report the number of studies excluded at each stage of screening | Yes | Figure 1 |
|  |  | 19.3 | Report brief reasons for exclusion from the full text stage | Yes | Figure 1 |
|  |  | 19.4 | Present a Preferred Reporting Items for Systematic Reviews and Meta-Analyses (PRISMA)-like flowchart (www.prisma-statement.org). | Yes | Figure 1 |
| **Sample sizes and study characteristics** | **80%** | 20.1 | Report the number of studies and effect sizes for data included in meta-analyses | Yes | Pages 29-33 |
|  |  | 20.2 | Report the number of studies and effect sizes for subsets of data included in meta-regressions | Yes | Pages 29-33 |
|  |  | 20.3 | Provide a summary of key characteristics for reported outcomes (either in text or figures; e.g. one quarter of effect sizes reported for vertebrates and the rest invertebrates) | Yes | Pages 29-33 |
|  |  | 20.4 | Provide a summary of limitations of included moderators (e.g. collinearity and overlap between moderators) | Yes | Pages 29-33 |
|  |  | 20.5 | Provide a summary of characteristics related to individual study quality (risk of bias) | No | n/a |
| **Meta-analysis** | **100%** | 21.1 | Provide a quantitative synthesis of results across studies, including estimates for the mean effect size, with confidence/credible intervals | Yes | Pages 29-33 |
| **Heterogeneity** | **100%** | 22.1 | Report indicators of heterogeneity in the estimated effect (e.g. *I*^2^, tau^2^ and other variance components) | Yes | Pages 29-33 |
| **Meta-regression** | **50%** | 23.1 | Provide estimates of meta-regression slopes (i.e. regression coefficients) and confidence/credible intervals | Yes | Pages 30-34 |
|  |  | 23.2 | Include estimates and confidence/credible intervals for all moderator variables that were assessed (i.e. complete reporting) | Yes | Tables S2-S4 |
|  |  | 23.3 | Report interactions, if they were included | No | n/a |
|  |  | 23.4 | Describe outcomes from model selection, if done (e.g. *R*^2^ and AIC) | No | n/a |
| **Outcomes of publication bias and sensitivity analysis** | **100%** | 24.1 | Provide results for the assessments of the risks of bias (e.g. Egger's regression, funnel plots) | Yes | Pages 29-33 |
|  |  | 24.2 | Provide results for the robustness of the review's results (e.g. subgroup analyses, meta-regression of study quality, results from alternative methods of analysis, and temporal trends) | Yes | Pages 30-33 |
| **Discussion** | **100%** | 25.1 | Summarise the main findings in terms of the magnitude of effect | Yes | Pages 33-34 |
|  |  | 25.2 | Summarise the main findings in terms of the precision of effects (e.g. size of confidence intervals, statistical significance) | Yes | Pages 33-34 |
|  |  | 25.3 | Summarise the main findings in terms of their heterogeneity | Yes | Page 42 |
|  |  | 25.4 | Summarise the main findings in terms of their biological/practical relevance | Yes | Pages 33-36 |
|  |  | 25.5 | Compare results with previous reviews on the topic, if available | Yes | Page 33 |
|  |  | 25.6 | Consider limitations and their influence on the generality of conclusions, such as gaps in the available evidence (e.g. taxonomic and geographical research biases) | Yes | Pages 33-40 |
| **Contributions and funding** | **100%** | 26.1 | Provide names, affiliations, and funding sources of all co-authors | Yes | Pages 1, 41 |
|  |  | 26.2 | List the contributions of each co-author | Yes | Page 42 |
|  |  | 26.3 | Provide contact details for the corresponding author | Yes | Page 1 |
|  |  | 26.4 | Disclose any conflicts of interest | No | n/a |
| **References** | **100%** | 27.1 | Provide a reference list of all studies included in the systematic review or meta-analysis | Yes | Pages 42-51 |
|  |  | 27.2 | List included studies as referenced sources (e.g. rather than listing them in a table or supplement) | Yes | Pages 42-51 |
