# Supplementary material for: Male alternative reproductive tactics and sperm competition: a meta‐analysis
Source: Biol Rev Camb Philos Soc. 2022 Feb 28;97(4):1365–88. doi: 10.1111/brv.12846 (PMC9541908; doi:10.1111/brv.12846)
Supplement: Supplementary file 2 — Fig. S1. PRISMA diagram summarising the literature search and study screening processes. Table S1. Methods for calculating the standardised mean difference (Hedges' d) and the location of the data collected. Fig. S2. Phylogenetic tree for the 53 species in the testis size data set. Fig. S3. Phylogenetic tree for the 32 species in the sperm quantity data set. Fig. S4. Phylogenetic tree for the 33 species in the sperm traits data set. Table S2. Sources used for the sneaker frequency data. Fig. S5. Histogram showing the distribution of sneaker frequency across 54 species. Fig. S6. Differences in testes size (Hedges' d) between male alternative reproductive tactics (ARTs) in relation to taxonomic group, after removing studies using the gonadosomatic index. Table S3. Mean effect size estimates (Hedges' d), 95% confidence intervals, and sample sizes for the testis size data set. Fig. S7. Relationship between effect size (Hedges' d) and publication year for the sperm quantity data set. Table S4. Mean effect size estimates (Hedges' d), 95% confidence intervals, and sample sizes for the sperm quantity data set. Table S5. Mean effect size estimates (Hedges' d), 95% confidence intervals, and sample sizes for the sperm traits data set. [file BRV-97-1365-s002.docx]

**Supplementary tables and figures:**

Male alternative reproductive tactics and sperm competition: a meta-analysis

Liam R. Dougherty^1*^, Michael J. A. Skirrow^2^, Michael D. Jennions^2^, and Leigh W. Simmons^3^

^1^*Department of Evolution, Ecology and Behaviour; University of Liverpool; Crown Street; Liverpool; L69 7RB; UK*

^2^*Evolution, Ecology and Genetics, Research School of Biology, The Australian National University, Canberra, ACT, 0200, Australia*

^3^*Centre for Evolutionary Biology, School of Biological Sciences, The University of Western Australia, Crawley, WA, 6009, Australia*

^*^E-mail: liam.dougherty@liverpool.ac.uk Tel: (+44) 0151 795 7771


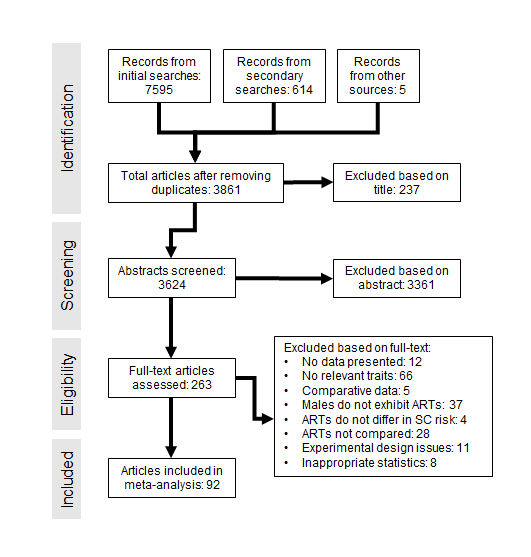


**Fig. S1.** PRISMA diagram summarising the literature search and study screening processes.

**Table S1.** Methods for calculating the standardised mean difference (Hedges’ *d*), and the location of the data collected. For the remaining three papers [Hettyey & Roberts (2005), Munguía-Steyer *et al*. (2012), and Smith (2012)] no directional effect size data was obtained.

| **Study** | **Method** | **Data location** |
| --- | --- | --- |
| Almeida et al. (2012) | Means and variances | Table 1 |
| Alonzo et al. (2016) | Means and variances | Figure 2 |
| Apostolico & Marian (2017) | Mann-Whitney U test | Table 1 |
| Apostolico & Marian (2018) | Means and variances | Text |
| Awata et al. (2006) | ANCOVA | Figure 1 |
| Awata et al. (2008) | ANCOVA | Figure 2 |
|  | Means and variances | Table 2 |
| Bartlett et al. (2017) | *t*-test | Supplementary data |
| Bleeker et al. (2017) | Mann-Whitney *U* test | Figure 3 |
| Burness et al. (2004) | *t*-test | Text |
|  | Means and variances | Figure 1 |
| Burness et al. (2005) | Means and variances | Figure 1 |
|  | *t*-test | Text |
| Butts et al. (2012) | *t*-test | Text |
|  | Means and variances | Text |
| Butts et al. (2017) | Means and variances | Figure 3 |
| Byrne (2004) | ANCOVA | Figure 1 |
|  | Means and variances | Figure 2 |
| Clotfelter et al. (2017) | Means and variances | Figures 4 & 5 |
| Cote et al. (2009) | Means and variances | Figure 1 & Table 1 |
| Fasel et al. (2017) | Means and variances | Figures 1 & 2 |
| Fitzpatrick et al. (2007) | Means and variances | Figure 1 & Table 2 |
| Fitzpatrick et al. (2016) | ANCOVA | Figure 2 |
|  | Means and variances | Figures 3 & 4 |
| Flannery et al. (2013) | Means and variances | Figure 2, text |
|  | ANCOVA | Text |
| Fletcher (1999) | Means and variances | Table 1 |
| Gage et al. (1995) | Mann-Whitney *U* test | Text |
|  | ANCOVA | Figure 2 |
|  | Means and variances | Text |
| Goncalves et al. (2008) | Mann-Whitney *U* test | Figure 2 |
| Green et al. (2020) | Mann-Whitney *U* test | Figure 2 |
| Hettyey & Roberts (2007) | Means and variances | Table 1 |
|  | ANCOVA | Figure 1a |
| Hurtado-Gonzales & Uy (2009) | ANCOVA | Figure 5 |
|  | Means and variances | Table 3 & Text |
| Iwata et al. (2011) | Means and variances | Text |
| Jennings & Philipp (1992) | *t*-test | Text |
| Katoh et al. (2005) | Means and variances | Figure 2b |
| Kelly (2008) | ANCOVA | Figure 2 |
|  | Means and variances | Text |
| Kortet et al. (2004) | Means and variances | Figure 1 |
| Koseki & Maekawa (2002) | Means and variances | Text |
| Kvarnemo et al. (2010) | ANCOVA | Figure 2c |
| Lara et al. (2020) | Means and variances | Figure 1 |
| Leach & Montgomerie (2000) | *t*-test | Text |
| Lenhert et al. (2017) | Means and variances | Text |
| Lewis & Pitcher (2017b) | Means and variances | Figure 2 |
| Lewis & Pitcher (2017a) | Means and variances | Figure 1 & Figure 2 |
| Locatello et al. (2007) | *t*-test | Text |
|  | Means and variances | Figure 1 & Figure 2 |
| Locatello et al. (2013) | Means and variances | Figure 1 |
| Loveland et al. (2021) | ANCOVA | Raw data |
| Makiguchi et al. (2016) | Means and variances | Figure 2 |
|  | ANCOVA | Raw data |
| Marentette et al. (2009) | Means and variances | Figure 4 & Table 1 |
| Mazzoldi et al. (2000) | Means and variances | Table 3 |
| Meniri et al. (2019) | Means and variances | Table 2 |
| Miller et al. (2019) | Mann-Whitney *U* test | Figure 1b |
| Nakanishi & Takegaki (2019) | Mann-Whitney *U* test | Figure 2 |
|  | *t*-test | Text |
|  | Means and variances | Figure 3 |
| Neat (2001) | Means and variances | Figure 5 |
|  | *t*-test | Text |
| Neat et al. (2003) | Means and variances | Table 1 |
| Neff et al. (2003) | Means and variances | Figure 3 |
| Olsson et al. (2009) | Means and variances | Text |
| Ota & Kohda (2006) | Means and variances | Figure 3 |
| Ota et al. (2010) | Means and variances | Figure 1 |
| Ota et al. (2011) | ANCOVA | Figure 2 |
| Ota et al. (2014a) | ANCOVA | Figure 5 |
| Ota et al. (2014b) | Means and variances | Supplementary data |
| Peer et al. (2000) | Means and variances | Table 1 |
| Pilastro & Bisazza (1999) | Mann-Whitney *U* test | Text |
| Poli et al. (2018) | Means and variances | Figure 1 |
| Pujolar et al. (2012) | ANCOVA | Figure 1 |
| Rasotto & Mazzoldi (2002) | Means and variances | Figure 2, Table 2 |
| Rosa et al. (2019) | Means and variances | Figures 3 & 4 |
| Rowe et al. (2010) | Means and variances | Table 1 |
| Rudolfsen et al. (2006) | Means and variances | Figures 1, 3 & 4 |
| Saraiva et al. (2010) | Means and variances | Table 1 |
| Sasson et al. (2015) | Means and variances | Text |
| Sato et al. (2004) | Means and variances | Table 1 |
| Scharer & Robertson (1999) | Means and variances | Text |
| Schrempf et al. (2016) | Means and variances | Text |
| Schutz et al. (2010) | Means and variances | Text |
| Simmons & Buzatto (2014) | Means and variances | Figure 2 |
| Simmons et al. (1999) | Means and variances | Text |
| Simmons et al. (2007) | ANCOVA | Raw data |
| Smith & Reichard (2013) | Paired *t*-test | Text |
| Smith & Ryan (2010) | Means and variances | Table 1 |
|  | ANCOVA | Figure 1 |
| Stockley et al. (1994) | Means and variances | Table 1 |
| Stoltz & Neff (2006) | Mann-Whitney *U* test | Raw data |
|  | Means and variances | Text |
| Taborsky et al. (2018) | Mann-Whitney *U* test | Figure 3 |
|  | Means and variances | Figure 2, text |
| Tomkins & Simmons (2002) | ANCOVA | Figure 1d |
| Uglem et al. (2000) | Means and variances | Figure 4 |
| Uglem et al. (2001) | *t*-test | Text |
|  | Mann-Whitney *U* test | Text |
| Uglem et al. (2002) | Means and variances | Table 1 |
|  | *t*-test | Text |
|  | Means and variances | Text |
| Vladic (2000) | Mann-Whitney *U* test | Figure 2a |
|  | Means and variances | Figure 1 |
| Vladic (2006) | Means and variances | Table 2 |
| Vladic & Jarvi (2001) | Means and variances | Table 1 |
| Vladic et al. (2002) | Means and variances | Table 1, text |
| Vladic et al. (2010) | Means and variances | Table 1 |
| Warner & Lejeune (1985) | Means and variances | Table 2 |
| Yamamoto et al. (2015) | Mann-Whitney *U* test | Figure 2 |
|  | Means and variances | Figure 2, Table 1 |
| Young et al. (2013) | Means and variances | Raw data |


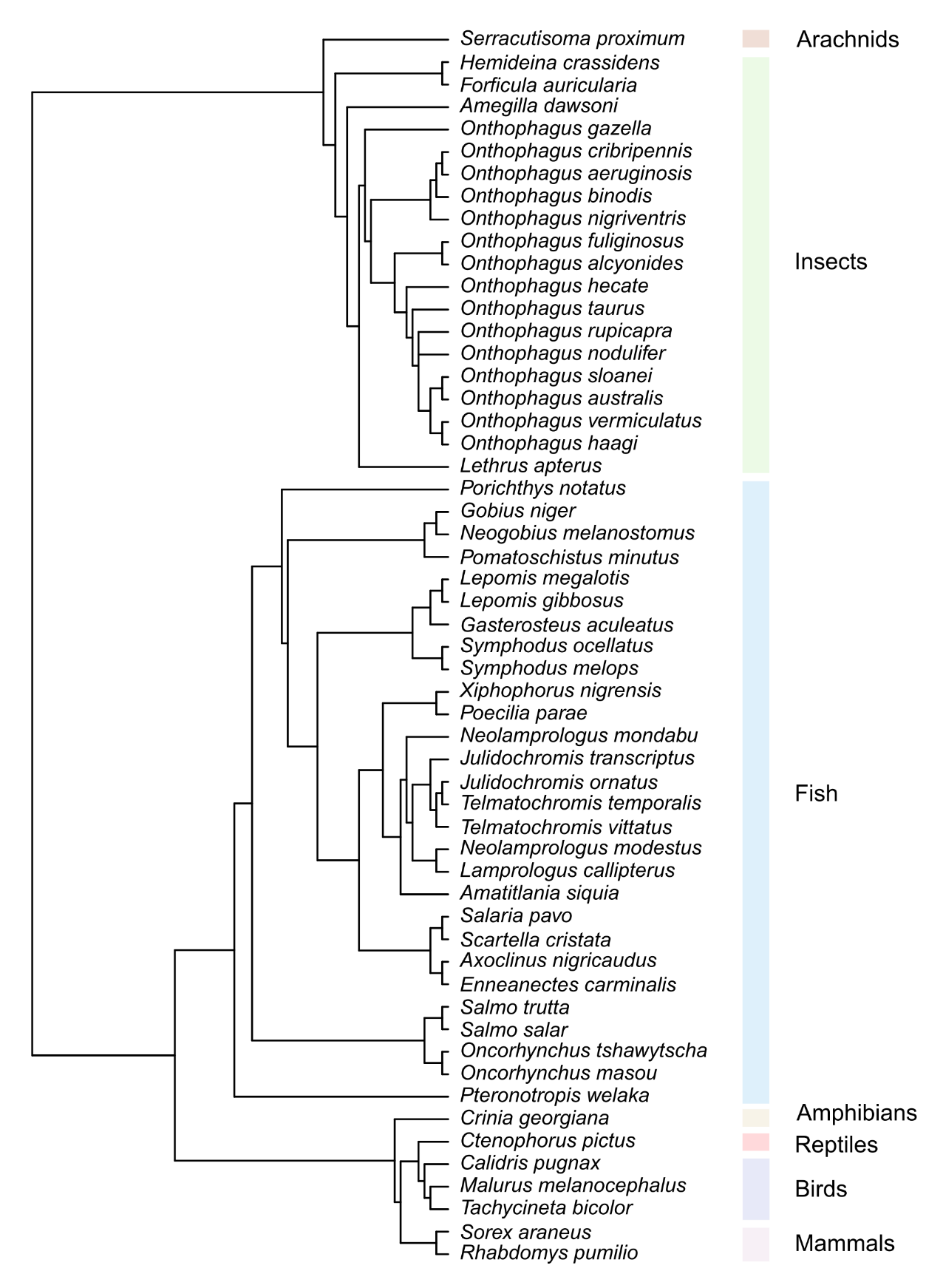


**Fig. S2.** Phylogenetic tree for the 53 species in the testis size data set. Note that the branch lengths are not time-calibrated.


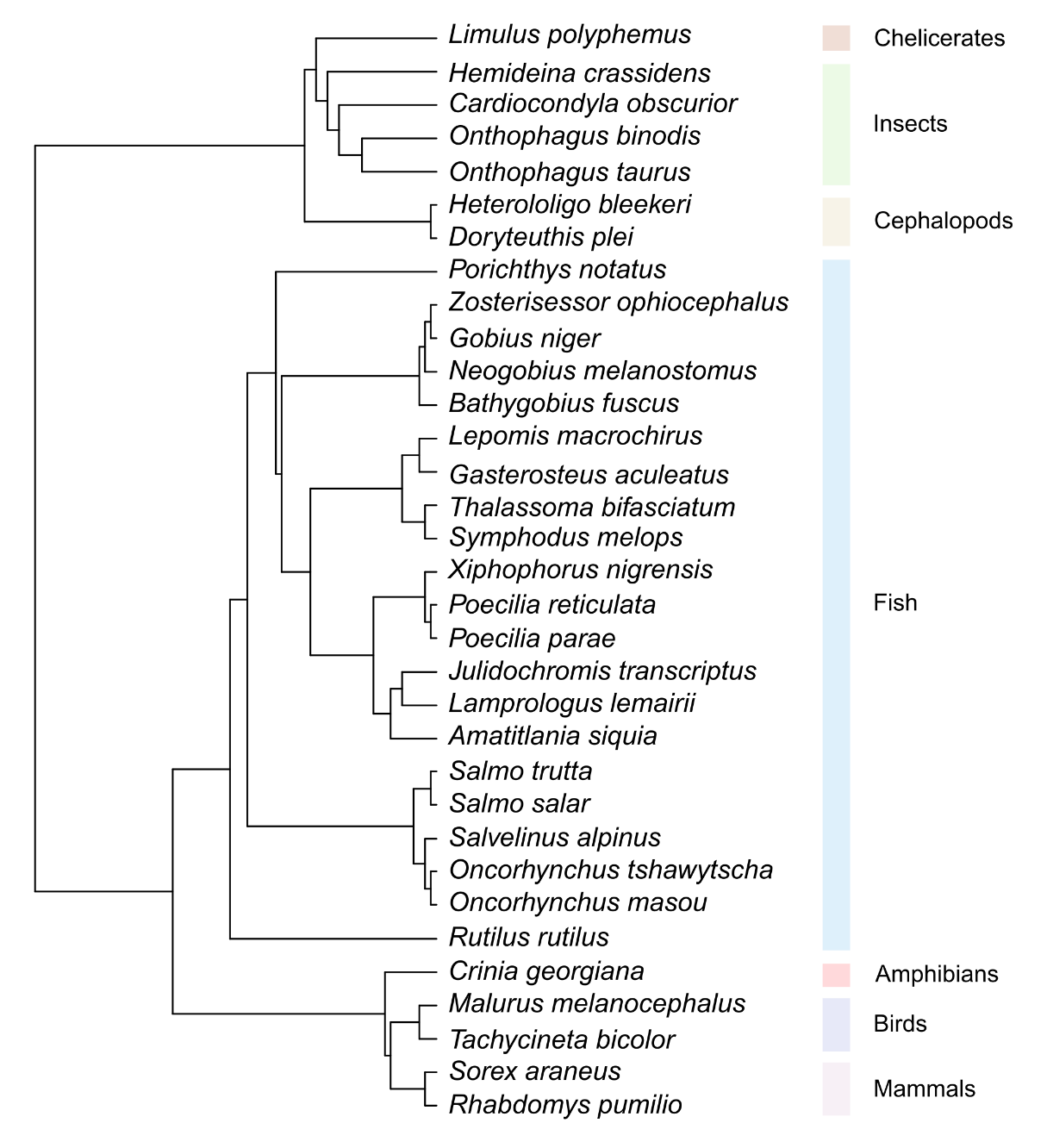


**Fig. S3.** Phylogenetic tree for the 32 species in the sperm quantity data set. Note that the branch lengths are not time-calibrated.


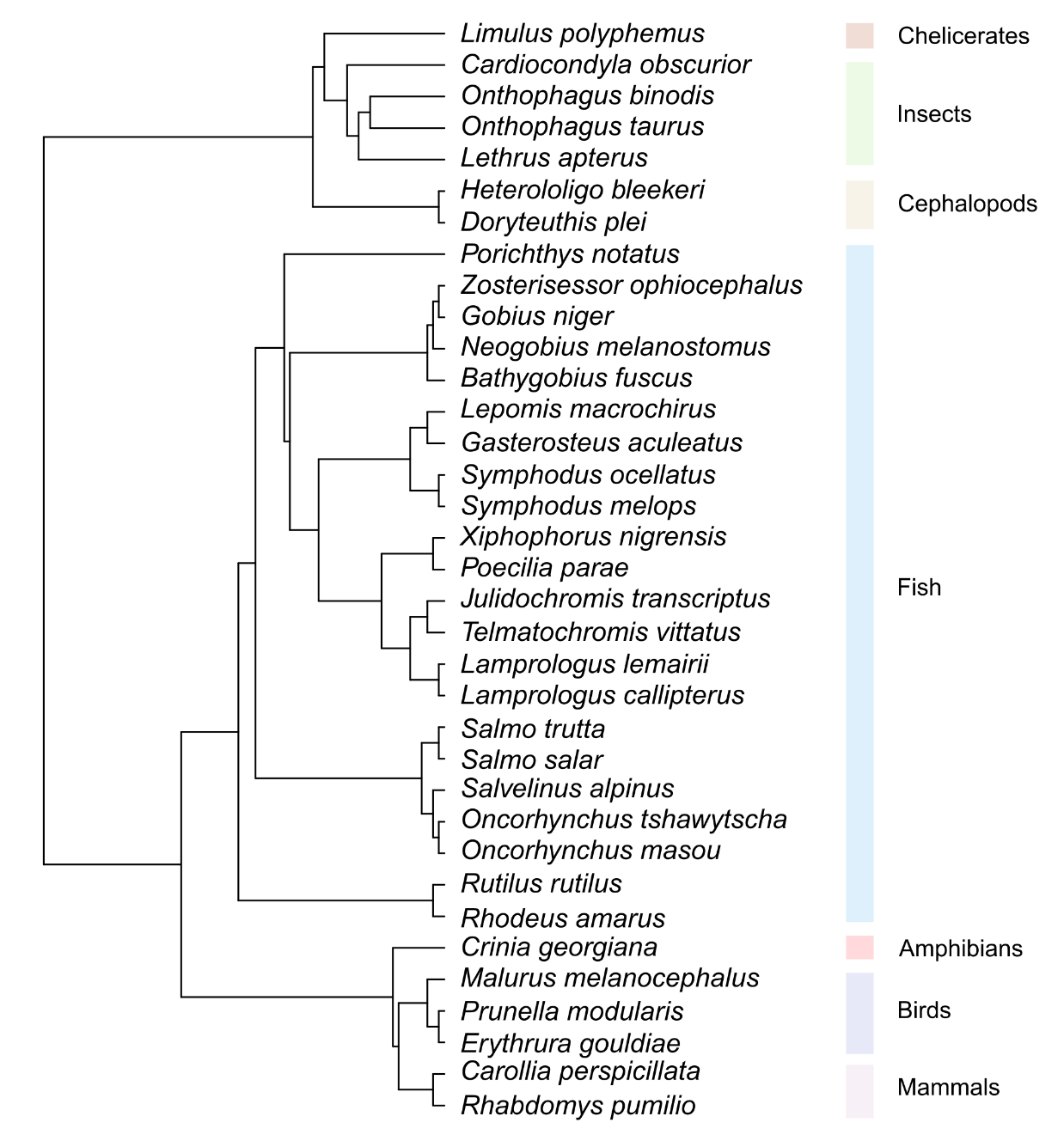


**Fig. S4.** Phylogenetic tree for the 33 species in the sperm traits data set. Note that the branch lengths are not time-calibrated.

**Table S2.** Sources used for the sneaker frequency data. See main text for references.

| **Species** | **Strategies** | **Sneaker frequency** | **Source** |
| --- | --- | --- | --- |
| Amatitlania siquia | Parental *vs* sneaker | 0.02 | Clotfelter *et al*. (2017) |
| Axoclinus nigricaudus | Territorial *vs* sneaker | 0.76 | Neat (2001) |
| Bathygobius fuscus | Nesting *vs* sneaker | 0.87 | Takegaki *et al*. (2012) |
| Carollia perspicillata | Harem *vs* sneaker | 0.8 | Fasel *et al*. (2017) |
| Ctenophorus pictus | Territorial *vs* sneaker | 0.39 | Olsson *et al*. (2007) |
| Doryteuthis plei | Guarder *vs* sneaker | 0.19 | Iwata & Sakurai (2007) |
| Enneanectes carminalis | Territorial *vs* sneaker | 0.39 | Neat (2001) |
| Forficula auricularia | Guarder *vs* sneaker | 0.4 | Tomkins & Simmons (2002) |
| Gasterosteus aculeatus | Parental *vs* sneaker | 0.38 | Cote *et al*. (2009) |
| Gobius niger | Guarder *vs* sneaker | 0.4 | Rasotto & Mazzoldi (2002) |
| Hemideina crassidens | Guarder *vs* sneaker | 0.28 | Kelly (2005) |
| Heterololigo bleekeri | Guarder *vs* sneaker | 0.27 | Iwata *et al*. (2011) |
| Julidochromis ornatus | Breeder *vs* cooperative breeder | 0.15 | Awata *et al*. (2006) |
| Lamprologus callipterus | Nesting *vs* sneaker | 0.51 | Sato *et al*. (2004) |
| Lamprologus lemairii | Nesting *vs* sneaker | 0.5 | Ota *et al*. (2014*b*) |
| Lepomis gibbosus | Parental *vs* sneaker | 0.54 | Almeida *et al*. (2012) |
| Lepomis macrochirus | Parental *vs* sneaker | 0.53 | Gross (1982) |
| Lepomis megalotis | Guarder *vs* sneaker | 0.26 | Jennings & Philipp (1992) |
| Lethrus apterus | Guarder *vs* sneaker | 0.14 | Rosa *et al*. (2019) |
| Limulus polyphemus | Guarder *vs* satellite | 0.66 | Brockmann (1990) |
| Malurus melanocephalus | Breeder *vs* helper | 0.18 | Rowe *et al*. (2010) |
| Neogobius melanostomus | Parental *vs* sneaker | 0.5 | Marentette *et al*. (2009) |
| Neolamprologus mondabu | Harem *vs* sneaker | 0.14 | Ota *et al*. (2014*a*) |
| Oncorhynchus masou | Guarder *vs* sneaker | 0.86 | Koseki & Maekawa (2002) |
| Oncorhynchus tschwaytscha | Guarder *vs* sneaker | 0.29 | Flannery *et al*. (2013) |
| Onthophagus aeruginosis | Guarder *vs* sneaker | 0.2 | Simmons *et al*. (2007) |
| Onthophagus alcyonides | Guarder *vs* sneaker | 0.17 | Simmons *et al*. (2007) |
| Onthophagus australis | Guarder *vs* sneaker | 0.47 | Simmons *et al*. (2007) |
| Onthophagus binodis | Guarder *vs* sneaker | 0.3 | Simmons *et al*. (2007) |
| Onthophagus cribripennis | Guarder *vs* sneaker | 0.45 | Simmons *et al*. (2007) |
| Onthophagus fuliginosus | Guarder *vs* sneaker | 0.41 | Simmons *et al*. (2007) |
| Onthophagus gazella | Guarder *vs* sneaker | 0.34 | Simmons *et al*. (2007) |
| Onthophagus haagi | Guarder *vs* sneaker | 0.32 | Simmons *et al*. (2007) |
| Onthophagus hecate | Guarder *vs* sneaker | 0.29 | Simmons *et al*. (2007) |
| Onthophagus nigriventris | Guarder *vs* sneaker | 0.48 | Simmons *et al*. (2007) |
| Onthophagus nodulifer | Guarder *vs* sneaker | 0.21 | Simmons *et al*. (2007) |
| Onthophagus rupicapra | Guarder *vs* sneaker | 0.61 | Simmons *et al*. (2007) |
| Onthophagus sloanei | Guarder *vs* sneaker | 0.17 | Simmons *et al*. (2007) |
| Onthophagus taurus | Guarder *vs* sneaker | 0.6 | Simmons *et al*. (2007) |
| Onthophagus vermiculatus | Guarder *vs* sneaker | 0.21 | Simmons *et al*. (2007) |
| Pomatoschistus minutus | Nesting *vs* sneaker | 0.175 | Kvarnemo *et al*. (2010) |
| Porichthys notatus | Guarder *vs* sneaker | 0.07 | Fitzpatrick *et al*. (2016) |
| Pteronotropis welaka | Territorial *vs* non-territorial | 0.5 | Fletcher (1999) |
| Rutilus rutilus | Attractive *vs* unattractive | 0.5 | Kortet *et al*. (2004) |
| Salaria pavo | Guarder *vs* sneaker | 0.36 | Almada *et al*. (1994) |
| Scartella cristata | Nesting *vs* sneaker | 0.15 | Neat *et al*. (2003) |
| Serracutisoma proximum | Guarder *vs* sneaker | 0.1 | Munguía-Steyer *et al*. (2012) |
| Sorex araneus | Resident *vs* searcher | 0.5 | Stockley *et al*. (1994) |
| Symphodus melops | Nesting *vs* sneaker | 0.2 | Uglem *et al*. (2000) |
| Symphodus ocellatus | Nesting *vs* sneaker | 0.85 | Warner & Lejeune (1985) |
| Telmatochromis temporalis | Parental *vs* sneaker | 0.4 | Katoh *et al*. (2005) |
| Telmatochromis vittatus | Parental *vs* sneaker | 0.14 | Ota & Kohda (2006) |
| Thalassoma bifasciatum | Guarder *vs* sneaker | 0.77 | Warner & Robertson (1978) |
| Zosterisessor ophiocephalus | Guarder *vs* sneaker | 0.82 | Scaggiante *et al*. (1999) |


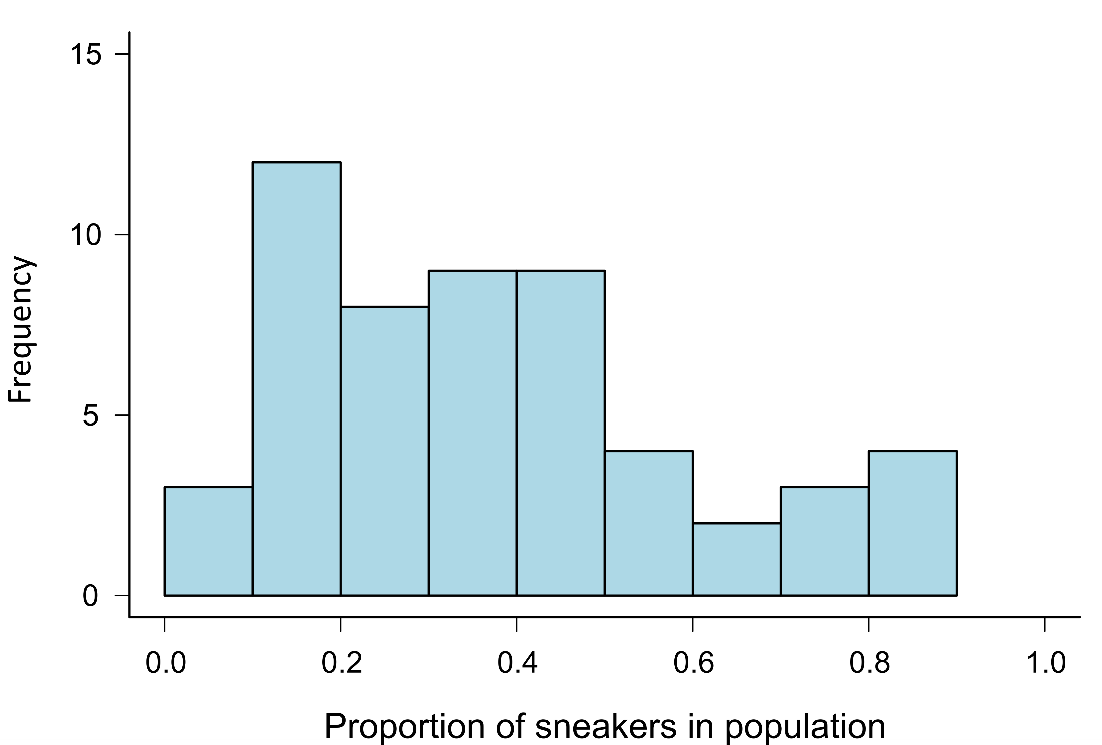


**Fig. S5.** Histogram showing the distribution of sneaker frequency across 54 species.


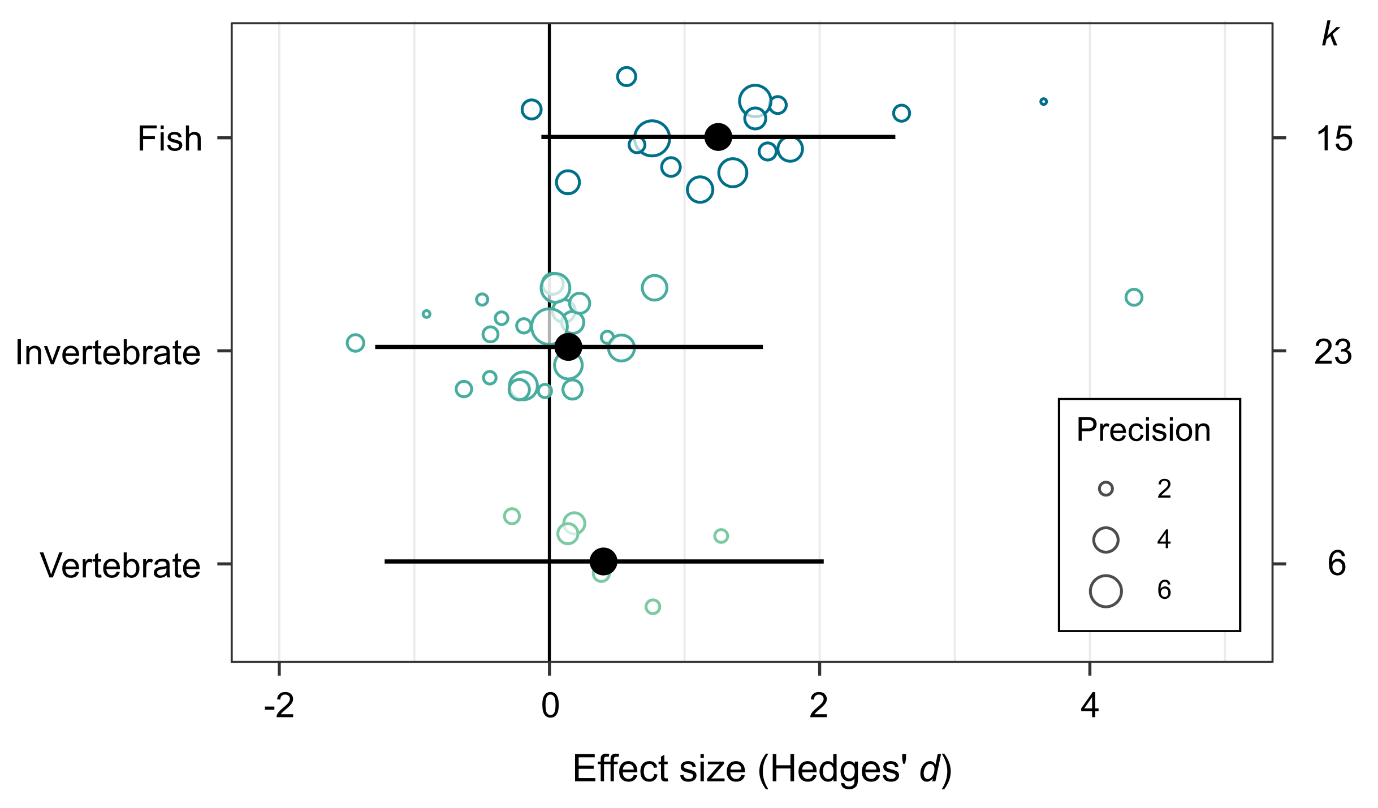


**Fig. S6.** Difference in testes size (Hedges’ *d*) between male alternative reproductive tactics (ARTs) in relation to taxonomic group, after removing studies using the gonadosomatic index. Points are scaled according to study variance (precision). Black points represent the meta-analytic mean, and black bars show the 95% confidence interval. *k* = number of effect sizes for each category.

**Table S3.** Mean effect size estimates (Hedges’ *d*), 95% confidence intervals, and sample sizes for the testes size data set. Means for the categorical moderator variables were obtained using a minus-intercept meta-regression, performed separately for each moderator. GSI, gonadosomatic index.

| **Factor** | **Level** | **Effect sizes** | **Studies** | **Species** | **Mean *d*** | **95% CI lower** | **95% CI upper** |
| --- | --- | --- | --- | --- | --- | --- | --- |
| All data |  | 74 | 51 | 53 | 0.87 | –0.16 | 1.90 |
| Directionless effect sizes removed |  | 73 | 50 | 52 | 0.90 | –0.15 | 1.95 |
| Polytomy removed |  | 72 | 51 | 51 | 0.87 | –0.15 | 1.89 |
| Taxonomic group | Fish | 44 | 37 | 28 | 1.82 | 1.37 | 2.27 |
|  | Invertebrate | 23 | 7 | 19 | 0.24 | –0.56 | 1.04 |
|  | Vertebrate | 7 | 7 | 6 | 0.19 | –0.84 | 1.21 |
| Mode of fertilisation | External | 44 | 37 | 27 | 1.84 | 1.39 | 2.29 |
|  | Internal | 30 | 14 | 26 | 0.24 | –0.36 | 0.84 |
| Tactic type | Fixed | 47 | 28 | 32 | 0.96 | –0.18 | 2.11 |
|  | Plastic | 1 | 1 | 1 | 0.00 | –2.87 | 2.87 |
|  | State-dependent | 26 | 22 | 20 | 0.73 | –0.53 | 1.99 |
| Measurement | GSI | 30 | 25 | 20 | 1.57 | 0.67 | 2.46 |
|  | Relative testes size | 41 | 23 | 34 | 0.58 | –0.24 | 1.39 |
|  | Absolute testes size | 3 | 3 | 3 | 0.80 | –0.96 | 2.56 |


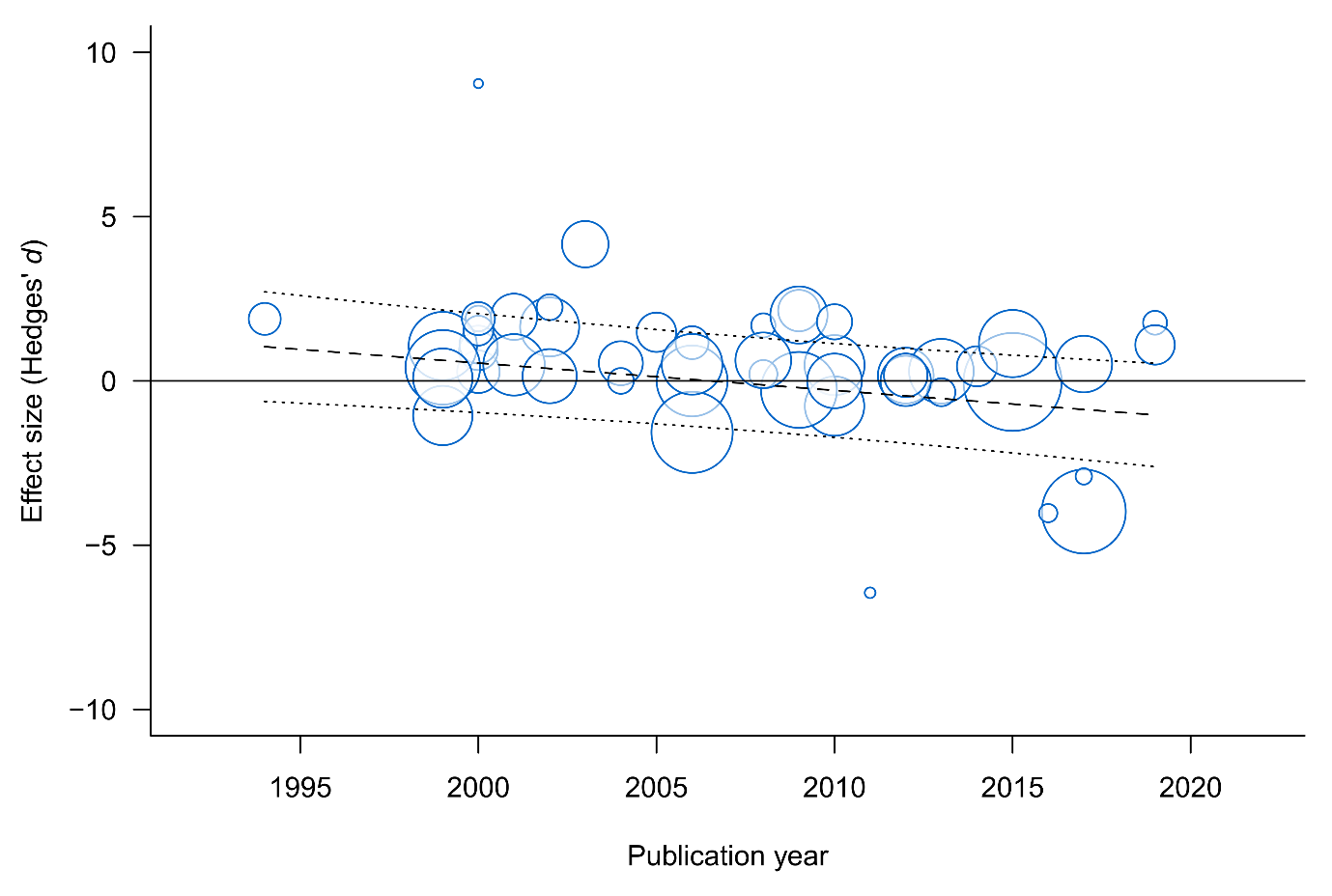


**Fig. S7.** Relationship between effect size (Hedges’ *d*) and publication year for the sperm quantity data set (*k* = 49). Each bubble represents an effect size, and bubble size is scaled to effect size precision (inverse standard error; larger bubbles reflect larger sample sizes). The

dashed line shows the predicted line from a meta-regression including study year as a covariate. Dotted lines show the 95% confidence intervals for the predicted line.

**Table S4.** Mean effect size estimates (Hedges’ *d*), 95% confidence intervals, and sample sizes for the sperm quantity data set. Means for the categorical moderator variables were obtained using a minus-intercept meta-regression, performed separately for each moderator.

| **Factor** | **Level** | **Effect sizes** | **Studies** | **Species** | **Mean *d*** | **95% CI lower** | **95% CI upper** |
| --- | --- | --- | --- | --- | --- | --- | --- |
| All data |  | 49 | 43 | 32 | –0.16 | –2.14 | 1.81 |
| Directionless effect sizes removed |  | 45 | 39 | 30 | –0.13 | –2.19 | 1.94 |
| Taxonomic group | Fish | 36 | 32 | 21 | 0.82 | –2.34 | 3.98 |
|  | Invertebrate | 7 | 6 | 7 | –1.42 | –4.98 | 2.14 |
|  | Vertebrate | 6 | 5 | 4 | 0.35 | –3.62 | 4.31 |
| Mode of fertilisation | External | 35 | 31 | 20 | –0.03 | –2.10 | 2.04 |
|  | Internal | 14 | 12 | 12 | –0.30 | –2.39 | 1.79 |
| Tactic type | Fixed | 27 | 24 | 15 | 0.12 | –2.17 | 2.41 |
|  | Plastic | 4 | 4 | 4 | –0.52 | –2.99 | 1.95 |
|  | State-dependent | 18 | 15 | 14 | –0.46 | –2.77 | 1.86 |
| Measurement | Sperm density | 20 | 19 | 14 | 0.44 | –0.52 | 1.39 |
|  | Sperm number | 13 | 13 | 12 | –0.44 | –1.41 | 0.54 |
|  | Ejaculate volume | 15 | 12 | 10 | 0.73 | –0.34 | 1.79 |
|  | Spermatophore size | 1 | 1 | 1 | –3.30 | –5.73 | –0.88 |
| Sperm allocation *vs* expenditure | Allocation | 7 | 6 | 7 | –0.69 | –2.79 | 1.41 |
|  | Expenditure | 42 | 37 | 26 | 0.09 | –1.86 | 2.04 |

**Table S5.** Mean effect size estimates (Hedges’ *d*), 95% confidence intervals, and sample sizes for the sperm traits data set. Means for the categorical moderator variables were obtained using a minus-intercept meta-regression, performed separately for each moderator.

| **Factor** | **Level** | **Effect sizes** | **Studies** | **Species** | **Mean *d*** | **95% CI lower** | **95% CI upper** |
| --- | --- | --- | --- | --- | --- | --- | --- |
| All data |  | 128 | 55 | 33 | 0.14 | –0.05 | 0.33 |
| Directionless effect sizes removed |  | 118 | 52 | 33 | 0.15 | –0.04 | 0.35 |
| Variance matrix (*r* = 0.25) |  | 128 | 55 | 33 | 0.14 | –0.07 | 0.34 |
| Variance matrix (*r* = 0.5) |  | 128 | 55 | 33 | 0.12 | –0.12 | 0.37 |
| Variance matrix (*r* = 0.75) |  | 128 | 55 | 33 | 0.12 | –0.16 | 0.38 |
| Taxonomic group | Fish | 102 | 43 | 22 | 0.14 | –0.40 | 0.68 |
|  | Invertebrate | 10 | 6 | 7 | 0.09 | –0.64 | 0.82 |
|  | Vertebrate | 16 | 6 | 4 | 0.05 | –0.70 | 0.80 |
| Mode of fertilisation | External | 103 | 43 | 22 | 0.08 | –0.26 | 0.43 |
|  | Internal | 25 | 12 | 11 | 0.19 | –0.22 | 0.60 |
| Tactic type | Fixed | 65 | 30 | 15 | 0.25 | 0.00 | 0.51 |
|  | Plastic | 7 | 4 | 4 | 0.08 | –0.52 | 0.67 |
|  | State-dependent | 56 | 21 | 15 | 0.05 | –0.21 | 0.30 |
| Trait | ATP content | 7 | 6 | 5 | 1.25 | 0.67 | 1.83 |
|  | Proportion of motile sperm | 28 | 25 | 17 | 0.21 | –0.06 | 0.48 |
|  | Sperm longevity | 21 | 19 | 14 | –0.11 | –0.44 | 0.22 |
|  | Sperm size | 29 | 25 | 22 | 0.05 | –0.22 | 0.32 |
|  | Sperm swimming speed | 43 | 36 | 22 | 0.16 | –0.08 | 0.40 |
